# The effectiveness and complexity of interventions targeting sedentary behaviour across the lifespan: a systematic review and meta-analysis

**DOI:** 10.1186/s12966-020-00957-0

**Published:** 2020-04-25

**Authors:** Nicole E. Blackburn, Jason J. Wilson, Ilona I. McMullan, Paolo Caserotti, Maria Giné-Garriga, Katharina Wirth, Laura Coll-Planas, Sergi Blancafort Alias, Marta Roqué, Manuela Deidda, Andrew T. Kunzmann, Dhayana Dallmeier, Mark A. Tully

**Affiliations:** 1 Centre for Health and Rehabilitation Technologies, Institute of Nursing and Health Research, School of Health Sciences, Ulster University, Newtownabbey, United Kingdom; 2 Department of Sports Science and Clinical Biomechanics, Center for Active and Healthy Ageing (CAHA), Syddansk Universitet, Odense, Denmark; 3 Department of Sport Sciences, Faculty of Psychology, Education and Sport Sciences Blanquerna, Universitat Ramon Llull, Barcelona, Spain; 4 School of Health and Life Sciences, Glasgow Caledonian University, Glasgow, United Kingdom; 5 Agaplesion Bethesda Clinic Ulm, Geriatric Centre Ulm/Alb-Donau, Ulm, Germany; 6 Department of Epidemiology and Medical Biometry, Ulm University, Ulm, Germany; 7 Fundació Salut i Envelliment–Universitat Autònoma de Barcelona, Barcelona, Spain; 8 Biomedical Research Institute Sant Pau (IIB-Sant Pau), Barcelona, Spain; 9 Health Economics and Health Technology Assessment (HEHTA), Institute of Health and Wellbeing (IHW), University of Glasgow, Glasgow, United Kingdom; 10 School of Medicine, Dentistry and Biomedical Sciences, Centre for Public Health, Queen’s University Belfast, Belfast, United Kingdom; 11 Department of Epidemiology, Boston University School of Public Health, Boston, USA; 12 Institute of Mental Health Sciences, School of Health Sciences, Ulster University, Newtownabbey, United Kingdom

**Keywords:** Sedentary behaviour, Systematic review, Meta-analysis, Complex interventions, Children, Adults

## Abstract

**Background:**

Evidence suggests that sedentary behaviour (SB) is associated with poor health outcomes. SB at any age may have significant consequences for health and well-being and interventions targeting SB are accumulating. Therefore, the need to review the effects of multicomponent, complex interventions that incorporate effective strategies to reduce SB are essential.

**Methods:**

A systematic review and meta-analysis were conducted investigating the impact of interventions targeting SB across the lifespan. Six databases were searched and two review authors independently screened studies for eligibility, completed data extraction and assessed the risk of bias and complexity of each of the included studies.

**Results:**

A total of 77 adult studies (*n*=62, RCTs) and 84 studies (*n*=62, RCTs) in children were included. The findings demonstrated that interventions in adults when compared to active controls resulted in non-significant reductions in SB, although when compared to inactive controls significant reductions were found in both the short (MD -56.86; 95%CI -74.10, -39.63; n=4632; I^2^ 83%) and medium-to-long term (MD -20.14; 95%CI -34.13, -6.16; n=4537; I^2^ 65%). The findings demonstrated that interventions in children when compared to active controls may lead to relevant reductions in daily sedentary time in the short-term (MD -59.90; 95%CI -102.16, -17.65; n=267; I^2^ 86%), while interventions in children when compared to inactive controls may lead to relevant reductions in the short-term (MD -25.86; 95%CI -40.77, -10.96; n=9480; I^2^ 98%) and medium-to-long term (MD -14.02; 95%CI -19.49, -8.55; n=41,138; I^2^ 98%). The assessment of complexity suggested that interventions may need to be suitably complex to address the challenges of a complex behaviour such as SB, but demonstrated that a higher complexity score is not necessarily associated with better outcomes in terms of sustained long-term changes.

**Conclusions:**

Interventions targeting reductions in SB have been shown to be successful, especially environmental interventions in both children and adults. More needs to be known about how best to optimise intervention effects. Future intervention studies should apply more rigorous methods to improve research quality, considering larger sample sizes, randomised controlled designs and valid and reliable measures of SB.

## Introduction

Sedentary behaviour (SB) is defined as any waking behaviour where the energy expenditure is low and the predominant posture is sitting, reclining or lying [1]. SB is a multi-faceted and complex behaviour which is accumulated in multiple domains such as work, school or home, during transport and leisure time [2]. Accumulating evidence suggests that SB is associated with poor health outcomes [3, 4]. These relationships appear to remain after statistical adjustment for physical activity (PA) levels. However, recent research in adults has indicated that MVPA can attenuate the risk of all-cause mortality of high levels of SB [5, 6]. There remains therefore some debate as to the independence or inter-dependence of these two behaviours [7]. SB is an established risk factor for cardiovascular disease, type 2 diabetes and all-cause mortality [8, 9], as well as an emerging risk factor for several cancers [10, 11]. In the UK, it has been estimated that chronic disease associated with SB costs the NHS £0.7bn per annum in direct healthcare costs [12].

Accordingly, many European countries have now incorporated recommendations to reduce SB and break up sitting time as part of their PA guidelines [13, 14]. The SB activities and contexts of primary concern include TV viewing and other screen-focused behaviours as well as prolonged sitting within domestic, school, workplace and transportation environments [15, 16]. Additionally, throughout the various stages of life, people spend time in different social (i.e. friends, students, colleagues, family) and organisational environments (i.e. school and work) [17], and so SB is age and life stage dependent. SB at any age may have significant consequences for health and well-being [18–20]. It is also influenced by multiple factors that operate at an individual, social and institutional level. Consequently, interventions should be context-specific and relevant to the population segments being targeted [17, 21], and the need to develop and evaluate behaviour-specific, multicomponent, complex interventions that incorporate effective strategies to reduce SB are essential.

A variety of strategies and frameworks have been applied to SB interventions including, individualised and community-based tailoring, incorporating environmental, behavioural or mixed approaches to reducing sitting time. Environmental interventions may aim to modify home, school and/or workplace layouts as well as restructuring outdoor spaces and/or facilities to reduce sedentary time [22, 23]. Behavioural interventions focus on theory driven approaches that have the potential to influence behavioural determinants to promote healthier behaviours [24, 25]. Mixed approach interventions can include a combination of both environmental and behavioural components [26].

While there is limited information about the minimal amount of SB change required to produce meaningful health benefits, a recent systematic review by Peachey *et al*. [27] suggested that a 30-minute per day reduction in SB could be an effective threshold for observing long-term health benefits, such as improving cardiometabolic risk biomarkers. One of the main challenges when assessing the effectiveness of interventions, is addressing the issues that arise when a range of approaches have been applied and a variety of components are included in the intervention design to improve the same outcome. The Medical Research Council (MRC) published an updated framework for the development and evaluation of complex interventions in 2019 [28]. Within this framework, one of the key considerations is understanding the range of effects and how they vary dependent on the context (i.e. among recipients, between sites and over time) and the causes of that variation (i.e. variability in individual level outcomes). In addition, the guidelines consider the active components (i.e. variants of a package of care) and complexity of the intervention and how they influence or impact the effect [28]. One of the novel aspects of this review is that, in addition to establishing effectiveness, this review aims to understand the causal mechanisms (i.e. something that can explain the observed effect) that produce effective outcomes (i.e. reduce SB) that can be applied to intervention development. Although a number of previous reviews have been conducted on the effectiveness of SB interventions, most focus on specific settings [23] or population groups [27, 29, 30]. Interventions in these reviews vary in their complexity, but this has not previously been investigated. There is therefore a need to comprehensively review the full range of interventions by both setting and target, taking into account both effectiveness and complexity, as these factors contribute to the scaling up of public health programmes to address SB in the population. Therefore, the aim of this systematic review was to synthesise and evaluate the effectiveness of SB interventional approaches to reduce sedentary time across the lifespan and establish the relationship between complexity and effectiveness.

## Methods

### Study Inclusion Criteria

The process of review was reported according to the PRISMA Statement guidelines [31]. Studies were eligible for inclusion if they met the following criteria: [1] any intervention of any length, frequency, and intensity targeting SB; [2] study designs with a control or comparison group (e.g. usual care, alternative intervention) where the primary aim was to change the SB of individuals assessed by self-report (e.g., questionnaires) or device-based measures (e.g., accelerometer data); [3] community-dwelling (i.e. not institutional care); [4] SB was a reported outcome and [5] published in a peer-reviewed English language journal.

According to our aim of assessing effectiveness of the interventions, all outcomes relating to SB (self-reported or device-based measures) such as sedentary time, leisure or occupational sitting time, transport time, screen, media or television time were included. For the assessment of the effectiveness of SB interventions, studies had to include a control group. As per Martin *et al.* [29], we included studies with any type of comparator, considering inactive controls such as; no intervention, waiting list, attention control (e.g. general health information), and usual care (e.g. general lifestyle counselling), as well as active comparisons against alternative treatment conditions (e.g. a structured exercise programme).

### Search Strategy

Relevant databases were searched using a search strategy adapted from previous reviews [29, 30]. A comprehensive search was performed up to 1 May 2019 using MEDLINE, PsycINFO, Web of Science, EMBASE, Physical Education Index, and SPORTDiscus. Keywords and title/abstract words related to exposure (sedentary lifestyle, sitting or lying, screen time, media time, driving) and intervention (intervention studies, health promotion, health education, behaviour change) were used. The search strategy was developed by authors (ATK, MAT and NEB) and is provided in Supplement A. Reference lists of the included studies and related systematic reviews were examined to identify any additional studies. Authors (ATK, NEB, MAT and JJW) independently reviewed titles and abstracts for inclusion. Two authors (NEB and MAT) then reviewed the full text of the remaining articles to determine final inclusion. All cases of disagreement were resolved by a third-party adjudicator, with included studies agreed by consensus.

### Data Extraction

Characteristics of the included studies were independently extracted by two authors (NEB, JJW, IIM, PC, MGG, LCP, KW, SBA and MAT) including: sample size, age of participants, study design, intervention type, setting, SB outcome, assessment tool, outcome measure, length of intervention, underlying behavioural theory, and details of the control or comparison group. SB data (mean, standard deviation (SD)) were extracted and entered into Review Manager (RevMan) (*Version 5.3. Copenhagen: The Nordic Cochrane Centre, The Cochrane Collaboration, 2014*).

### Risk of Bias

Authors independently assessed the risk of bias of each of the included studies using an adaptation of the Cochrane risk of bias tool [32]. Studies were appraised based on selection bias (i.e. random sequence generation, allocation concealment), detection bias (i.e. blinding of study personnel), attrition bias (high is less than 70% at follow-up) and the validity of the outcome measure included in the study (i.e. device-based versus self-reported measures). A judgement of ‘low risk’, ‘high risk’, or ‘unclear risk’ of bias was selected for each of the domains. We considered that studies had a high risk of bias when at least one of the criteria were judged as having a high risk of bias in any one of the criteria. Overall risk of bias was assessed as unclear if one or more of the criteria was assessed as unclear, but none were assessed as having a high risk of bias.

### GRADE Assessment

The GRADE (Grading of Recommendations, Assessment, Development and Evaluations) is a systematic framework developed by Cochrane for rating the certainty of evidence in systematic reviews and other evidence syntheses [33]. Two authors (NEB and IIM) independently assessed the quality of evidence using the Cochrane GRADE assessment tool. An overall GRADE quality rating was applied to a body of evidence across outcomes, usually by taking the lowest quality of evidence from all the outcomes that were critical to decision making. For each of risk of bias, imprecision, inconsistency, indirectness, and publication bias, authors had the option of decreasing their level of certainty one or two levels based on the evidence available for that outcome. All cases of disagreement were resolved by a third-party (MR), with the overall certainty of evidence agreed by consensus.

### Complexity Assessment

The Cochrane Collaboration’s intervention Complexity Assessment Tool for Systematic Reviews was used to assess the complexity of the included intervention studies [34]. The tool comprises ten dimensions and facilitates an in-depth, systematic assessment of the complexity of interventions. The level of complexity for each of the included studies was determined based on the assessment levels of the core and optional dimensions. Each dimension was graded as ‘simple’, ‘moderately complex’ or ‘complex’ based on the criteria for each of the ten components. Studies were grouped based on intervention type (i.e. behavioural, environmental or mixed) for interventions targeting adults (18 years and over) and children (aged under 18 years). The global score for each included study was calculated by the sum of the individual rating scores (simple = 1, moderately complex = 2, complex = 3). Two authors independently appraised intervention complexity of all included studies, with discrepancies resolved through discussion.

### Statistical Analysis

The included studies were grouped depending on intervention type (behavioural, environmental and mixed), length of follow-up (≤6 months, >6 months), age group of participants (children and adults), and control (active and inactive). A separate meta-analysis was performed based on the groupings listed above to calculate the pooled effect sizes for SB. The difference between the intervention groups and control/comparison group in the mean change from baseline to post-intervention and the comparison at follow-up were used as a measure of effect size. Separate meta-analyses were conducted for children and adults as we expected the type, context and outcomes of the interventions to differ based on their life stage. Where study authors reported multiple trial arms in a single trial, only the relevant arms were included. It was assumed that there could be much variation arising from the different populations and study designs. Therefore, prior to data synthesis, the clinical homogeneity with respect of the type of intervention, type of participants and the similarity of outcomes was assessed. Statistical heterogeneity of these groups of interventions was assessed using the I-squared statistic [32]. The meta-analyses were conducted in RevMan (Version *5.3. Copenhagen: The Nordic Cochrane Centre, The Cochrane Collaboration, 2014*).

The association between complexity and effectiveness was assessed through the examination of scatter plots where each studies global score for complexity was plotted against effect size (Cohen’s D). The strength of association between complexity and effectiveness was assessed using a Spearman’s rank order correlation test (IBM SPSS Statistics software, v23. Armonk, NY: IBM Corp) where *r*-value and significance (*p*-value) statistics were considered.

## Results

### Search Results

**Fig. 1:**
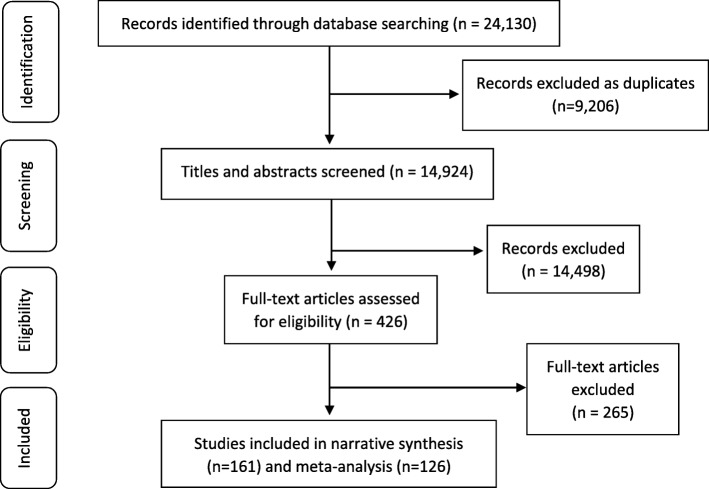
PRISMA flow diagram [31].

The search of the selected databases returned 24,130 potentially relevant studies, with 9,206 duplicates removed using Endnote (vX7.7.1. Toronto Canada: Thomson Reuters Cord, 2016). A total of 14,924 potentially relevant studies remained for title, abstract and key word screening. Following full-text screening and eligibility assessment of 426 studies, 161 studies were deemed as relevant and were included in the narrative synthesis, with 126 included in the meta-analysis. Figure 1 presents the identification, screening, eligibility and inclusion of studies within this systematic review [31].

### Included Studies

Within the 161 studies included in the narrative synthesis, 77 interventions [35–110] related to adults (mean age range: 26-76 years) while 84 interventions [64, 74, 108, 111–191] targeted children (mean age range: 2-19 years). The adult studies consisted of 62 randomised controlled trials (RCTs) of which nine were cluster RCTs and five were randomised cross-over trials. In addition, 15 studies employed a quasi-experimental design, of which thirteen controlled before and after trials, one was a natural experiment, and one an interrupted time series. The studies in children included 62 RCTs of which 23 were cluster RCTs and two were randomised cross-over trials. A further 22 employed a quasi-experimental design, of which 16 were controlled trials, five cluster controlled before and after trails, and one natural experiment. The control group participants received one of the following conditions: inactive (i.e. no intervention, waitlist control) or active (i.e. alternative intervention, generic health-related advice). Characteristics of the included studies are presented in Supplement B.

Most included studies measured SB using self-reported tools. Within the adult studies, over 10 different self-reported measures were used, including validated, and non-validated questionnaires. Within the studies in children, over 20 different self-reported measures were used, including validated questionnaires, non-validated parent-reported tools, and self-reported tools. The device-based measures used in adult and children studies included: ActivPAL, ActiGraph, and Hookie AM20. Additionally, adult studies included GENEactiv, MyWellness Key, and Sensewear armbands.

Within the meta-analysis, the follow-up length for 37 of the inactive control studies in adults was less than six months of which 20 were behavioural, 10 environmental, and seven were mixed interventions. The remaining 10 inactive control studies in adults included in the meta-analysis had a follow-up of more than six months of which included five mixed, four behavioural, and one environmental intervention. In 15 of the active control studies in adults, the follow-up was less than six months of which nine were behavioural, five mixed, and one environmental intervention. The remaining four active control studies in adults included in the meta-analysis had a follow-up of more than six months and were mixed interventions.

In 35 of the inactive control studies in children the follow-up was less than six months of which 18 were behavioural, nine were mixed, and eight environmental interventions. The remaining 24 inactive control studies in children included in the meta-analysis had a follow-up of more than six months of which included 16 mixed, six behavioural, and two environmental interventions. In 11 of the active control studies in children the follow-up was less than six months of which four were behavioural and seven were mixed interventions. The remaining three active control studies in children included in the meta-analysis had a follow-up of more than six months of which included two mixed and one behavioural intervention.

### Complexity Assessment

**Fig. 2:**
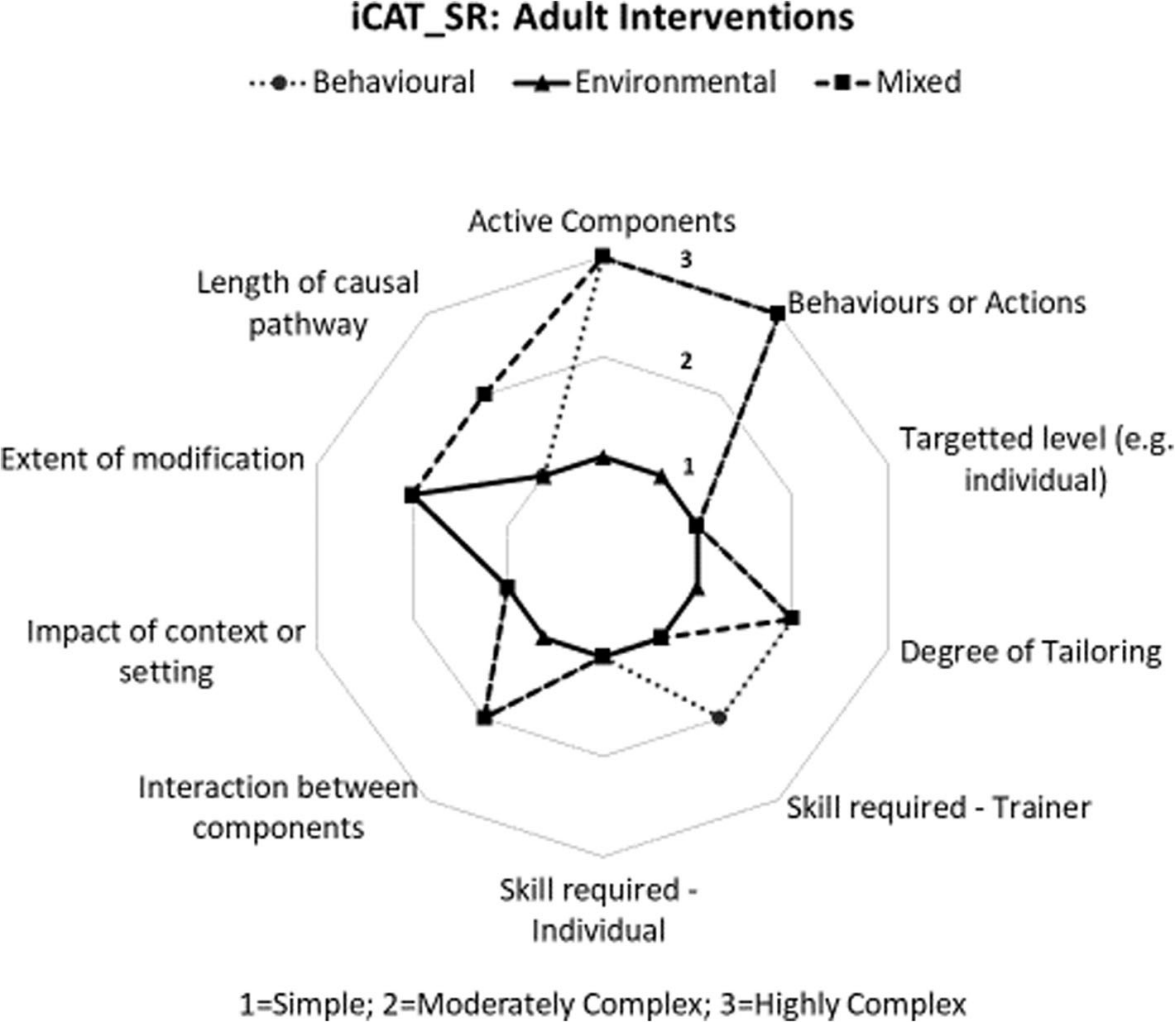
Complexity Score for Interventions in Adults [34].

**Fig. 3:**
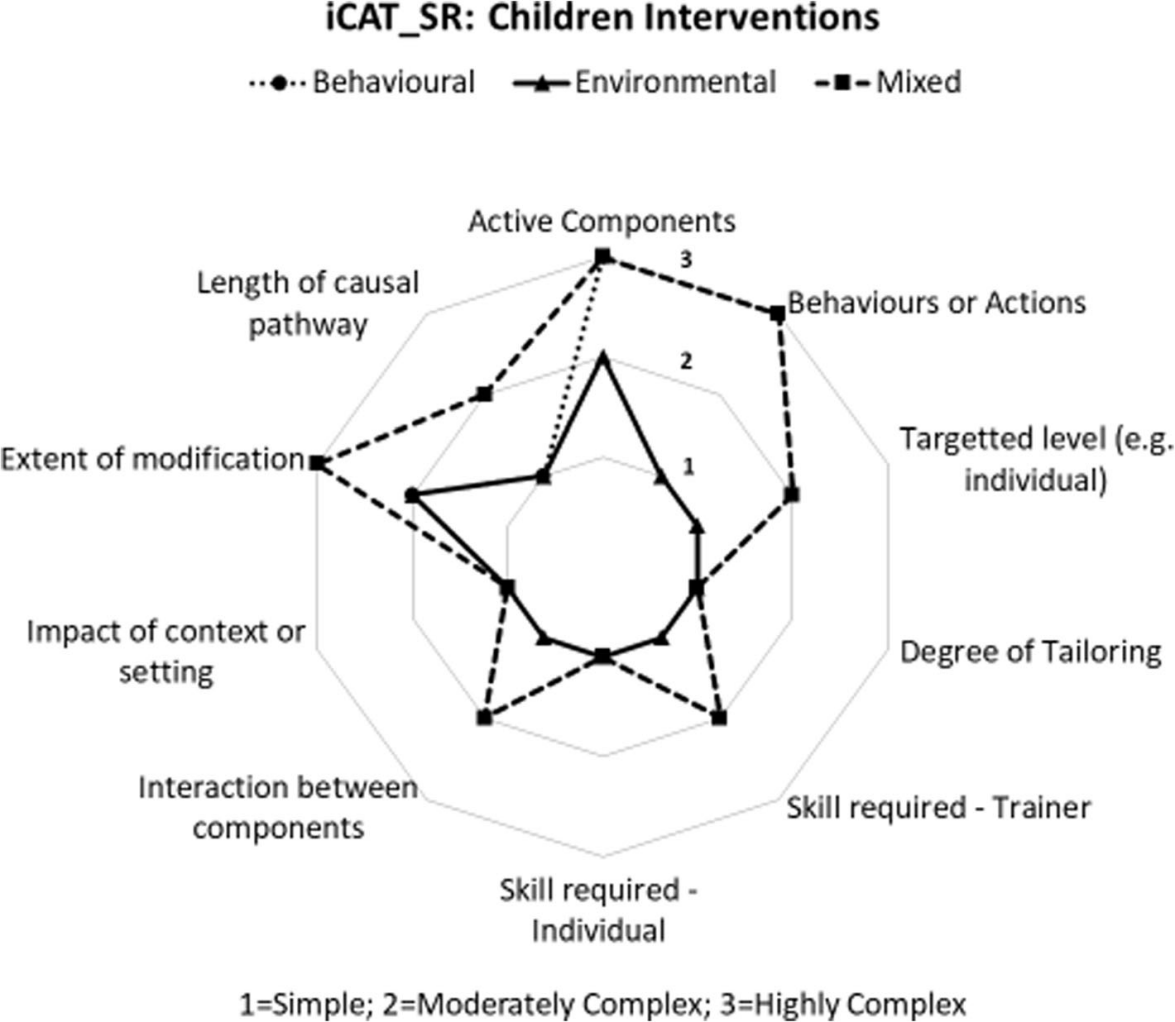
Complexity Score for Interventions in Children [34].

Each of the included studies were assessed for the level of complexity of the intervention components using the Cochrane Complexity Assessment Tool [34]. There was significant variety in the level of complexity of the included studies where global scores for complexity ranged from 10/30 (least complex) to 30/30 (most complex), with the mean score being 17.64 (SD 4.14). Results from the complexity assessment demonstrated that environmental interventions were less complex in both adult and children studies. In both adult and children studies, mixed and behavioural interventions scored as complex in relation to two dimensions including, the active components of the intervention and the behaviours or actions to which the intervention was directed. Mixed interventions carried out in children were scored as complex in relation to the degree to which the effects of the intervention were changed by recipient or provider factors. The complexity star chart for intervention studies in adults is presented in Figure 2 and the complexity star chart for intervention studies in children is presented in Figure 3.

**Fig. 4:**
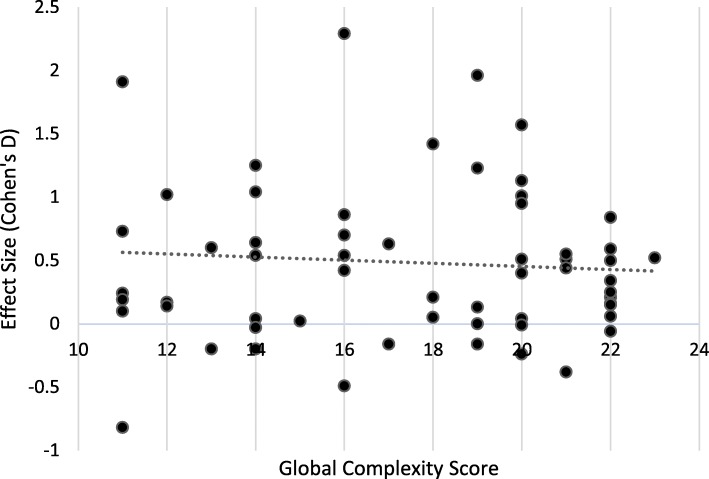
Relationship Between Complexity and Effectiveness of Interventions in Adults*

**Fig. 5:**
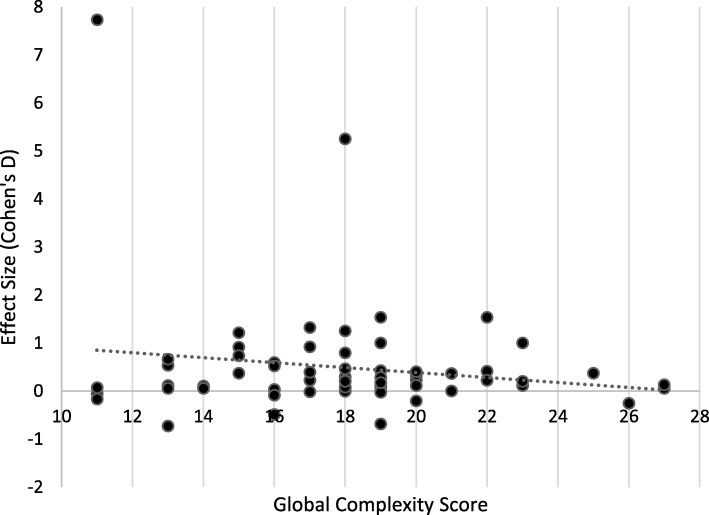
Relationship Between Complexity and Effectiveness of Interventions in Children**Line represents linear best fit line

The relationship between complexity and effectiveness by Cohen’s D effect size are presented in Figures 4 and 5. A Spearman’s rank-order correlation assessed the relationship, resulting in a non-significant relationship (*r*=-0.23; *p*=0.799), suggesting that for these interventions, there was no increased effectiveness with more complex interventions.

### Risk of Bias Assessment

The individual risk of bias assessment is included in Supplement C.

### Adult Interventions (n=77 studies)

**Fig. 6:**
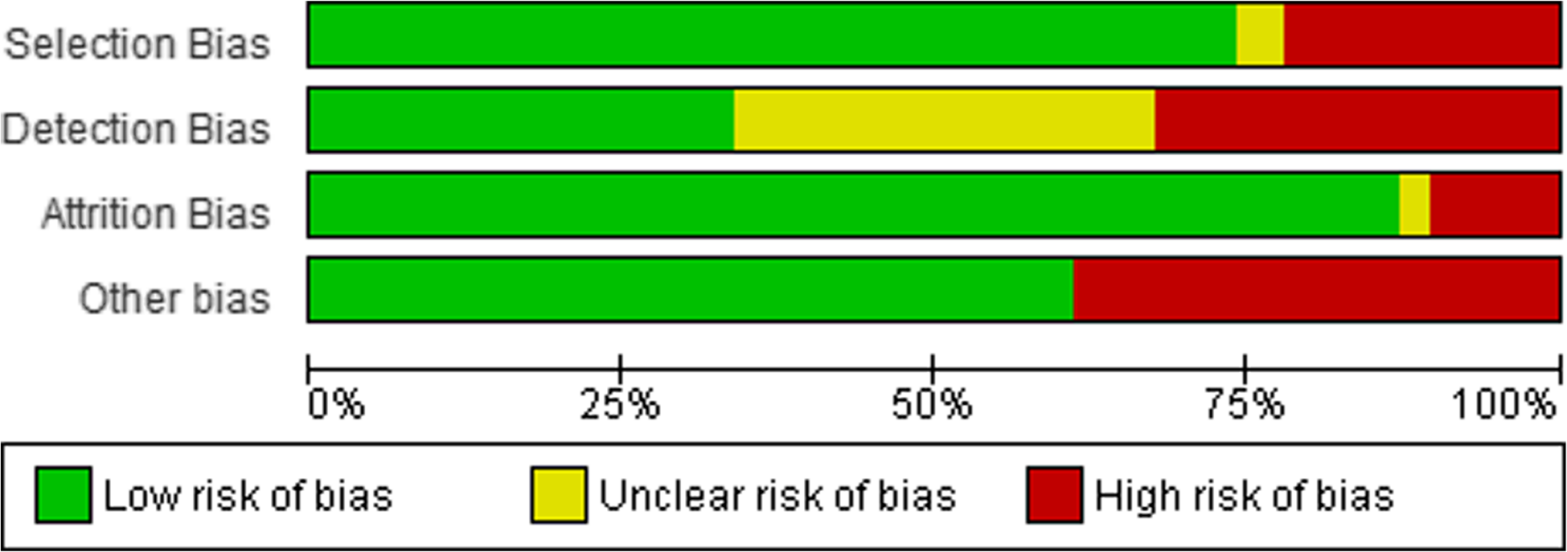
Risk of Bias - Interventions in Adults

Seventeen studies were rated as high risk for selection bias due to lack of detail regarding the randomisation process or because they were non-randomised. Three studies were rated as unclear due to a lack of information regarding how the random sequence was generated. Regarding detection bias, 26 studies did not report whether the study personnel were blinded to the intervention and were rated to be unclear risk for detection bias. Twenty-six studies were blinded and rated as low risk, with the remaining 25 studies rated as high risk as there was no allocation concealment. Eight studies were rated as a high risk of attrition bias, two studies were rated as unclear as they gave no indication of the number of participants returning for post-intervention follow-up. The validity of the outcome measure included in the study was also assessed for risk of bias. Thirty studies included self-reported measures of SB and were rated as high risk, with the remaining 47 studies rated as low risk due to the inclusion of device-based measures. The largest risk of bias for the adult interventions came from the validity of the outcome measure, with almost 40% of studies including self-reported measures of SB. A summary of the risk of bias assessment in adults is shown in Figure 6.

### Children Interventions (n=84 studies)

**Fig. 7:**
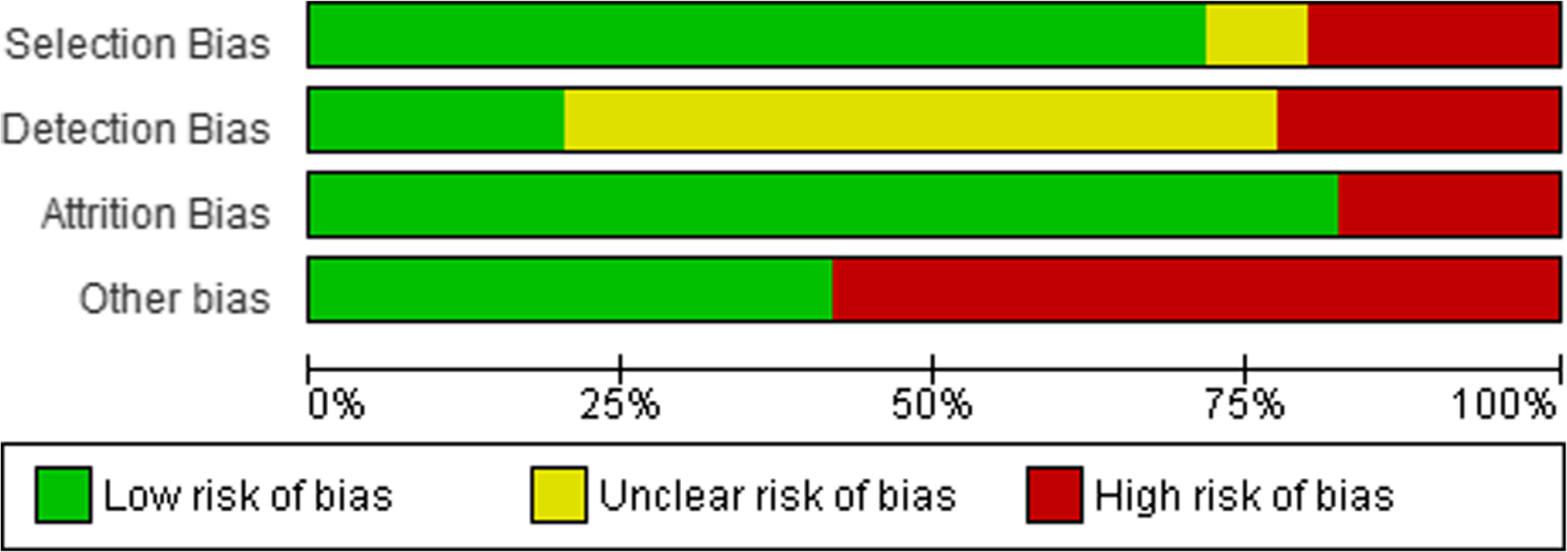
Risk of Bias - Interventions in Children

Seventeen studies were rated as high risk for selection bias as they were non-randomised. Seven studies were rated as unclear due to a lack of information regarding how the random sequence was generated. Regarding detection bias, 48 studies did not report whether the study personnel were blinded to the intervention and were rated to be unclear risk for detection bias. Seventeen studies were blinded and rated as low risk, with the remaining 19 studies rated as high risk as there was no allocation concealment. Fifteen studies were rated as high risk of attrition bias. The validity of the outcome measure included in the study was assessed for risk of bias. Forty-nine studies included self-reported measures and were rated as high risk, with the remaining 35 studies rated as low risk due to the inclusion of device-based measures. The largest risk of bias for the children interventions came from the validity of the outcome measure, with almost 60% of studies including self-reported measures of SB. A summary of the risk of bias assessment in children is shown in Figure 7.

### Effectiveness

Of the 161 studies included in the review, 126 studies provided data for inclusion in the meta-analysis. Studies were excluded from the meta-analysis due to insufficient data provided (i.e. did not provide a measure of variance) or the study’s SB outcome measures were inappropriate data types for meta-analyses.

### Effects of Adult Interventions

Studies in adults were pooled according to the type of intervention (i.e. behavioural, environmental or mixed), the length of follow up (≤6 months or >6 months) and type of control (active or inactive). The results from the meta-analysis of the adult studies is presented in Tables 1 and 2.

**Table 1:** Effectiveness of SB Interventions in Adults Compared to an Inactive Control

Subgroup 1: Behavioural	Studies (***n***=)	I (***n***=)	C (***n***=)	I^**2**^	MD (95%CI)
Outcome 1: SB min/day (≤6mo)	19	1179	1015	83%	-60.57 [-86.67, -34.46]
Outcome 2: SB min/day (>6mo)	4	701	501	63%	-7.30 [-28.98, 14.38]
Outcome 3: SB percentage (≤6mo)	1	56	63	N/A	-1.02 [-3.03, 0.99]
**Subgroup 2: Environmental**	**Studies (*n*=)**	**I (n=)**	**C (n=)**	**I**^**2**^	**MD (95%CI)**
Outcome 1: SB min/day (≤6mo)	8	181	167	86%	-64.05 [-104.64, -23.46]
Outcome 2: SB min/day (>6mo)	1	222	264	N/A	-35.20 [-80.94, 10.54]
Outcome 3: SB % (≤6mo)	2	52	49	0%	-13.95 [-15.33, -12.56]
**Subgroup 3: Mixed**	**Studies (n=)**	**I (n=)**	**C (n=)**	**I**^**2**^	**MD (95%CI)**
Outcome 1: SB min/day (≤6mo)	7	552	526	87%	-42.63 [-77.62, -7.63]
Outcome 2: SB min/day (>6mo)	5	1797	1052	72%	-30.25 [-52.92, -7.58]
**Overall Results**	**Studies (n=)**	**I (n=)**	**C (n=)**	**I**^**2**^	**MD (95%CI)**
Outcome 1: SB min/day (≤6mo)	34	1912	1708	83%	-56.86 [-74.10, -39.63]
Outcome 2: SB min/day (>6mo)	10	2720	1817	65%	-20.14 [-34.13, -6.16]
Outcome 3: SB % (≤6mo)	3	108	112	98%	-9.80 [-10.94, -8.65]

**Table 2:** Effectiveness of SB Interventions in Adults Compared to an Active Control

Subgroup 1: Behavioural	Studies (n=)	I (n=)	C (n=)	I^**2**^	MD (95%CI)
Outcome 1: SB min/day (≤6mo)	9	404	384	87%	-36.69 [-76.23, 2.86]
Outcome 2: SB min/day (>6mo)	4	145	152	69%	-3.77 [-20.95, 13.41]
**Subgroup 2: Environmental**	**Studies (n=)**	**I (n=)**	**C (n=)**	**I**^**2**^	**MD (95%CI)**
Outcome 1: SB min/day (≤6mo)	1	15	16	N/A	2.58 [-57.81, 62.97]
**Subgroup 3: Mixed**	**Studies(n=)**	**I(n=)**	**C(n=)**	**I**^**2**^	**MD (95%CI)**
Outcome 1: SB min/day (≤6mo)	3	88	97	0%	-11.13 [-35.60, 13.33]
Outcome 2: SB min/day (>6mo)	1	24	12	N/A	-29.60 [-72.36, 13.16]
Outcome 3: SB percentage (≤6mo)	2	35	36	0%	-1.29 [-4.01, 1.43]
**Overall Results**	**Studies (n=)**	**I (n=)**	**C (n=)**	**I**^**2**^	**MD (95%CI)**
Outcome 1: SB min/day (≤6mo)	13	507	497	81%	-25.89 [-53.51, 1.73]
Outcome 2: SB min/day (>6mo)	5	169	164	62%	-6.49 [-22.34, 9.37]
Outcome 3: SB percentage (≤6mo)	2	35	36	0%	-1.29 [-4.01, 1.43]

The findings from the adult interventions that included an inactive control demonstrated a clinically meaningful effect in the short-term i.e. ≤6 months [24]. The findings from the environmental and mixed interventions in the medium-term demonstrated a moderate but relevant reduction in daily SB. The results from the adult studies demonstrated moderate to high heterogeneity at both short and medium-term across both active and inactive control comparators. Results for the reductions in percentage of daily time in SB were not robust due to the scarcity of data.

### Effects of Children Interventions

Studies in children were pooled according to the type of intervention (i.e. behavioural, environmental or mixed), the length of follow up (≤6 months or >6 months) and type of control (active or inactive). The results from the meta-analysis of the children studies is presented in Tables 3 and 4.

**Table 3:** Effectiveness of SB Interventions in Children Compared to an Inactive Control

Subgroup 1: Behavioural	Studies (***n***=)	I (***n***=)	C (***n***=)	I^**2**^	MD (95%CI)
Outcome 1: SB min/day (≤6mo)	18	2490	2632	99%	-27.20 [-46.99, -7.40]
Outcome 2: SB min/day (>6mo)	6	1629	1567	87%	-6.20 [-26.42, 14.02]
**Subgroup 2: Environmental**	**Studies (*n*=)**	**I (*n*=)**	**C (*n*=)**	**I**^**2**^	**MD (95%CI)**
Outcome 1: SB min/day (≤6mo)	8	255	237	77%	-18.57 [-40.12, 2.99]
Outcome 2: SB min/day (>6mo)	2	41	41	0%	-8.75 [-17.18, -0.32]
**Subgroup 3: Mixed**	**Studies (*n*=)**	**I (*n*=)**	**C (*n*=)**	**I**^**2**^	**MD (95%CI)**
Outcome 1: SB min/day (≤6mo)	6	1941	1925	37%	-27.41 [-51.18, -3.63]
Outcome 2: SB min/day (>6mo)	15	18710	19150	99%	-17.54 [-24.15, -10.92]
Outcome 3: SB % (≤6mo)	3	627	435	0%	-1.70 [-2.93, -0.48]
**Overall Results**	**Studies (*n*=)**	**I (*n*=)**	**C (*n*=)**	**I**^**2**^	**MD (95%CI)**
Outcome 1: SB min/day (≤6mo)	32	4686	4794	98%	-25.86 [-40.77, -10.96]
Outcome 2: SB min/day (>6mo)	23	20380	20758	98%	-14.02 [-19.49, -8.55]
Outcome 3: SB % (≤6mo)	3	627	435	0%	-1.70 [-2.93, -0.48]

**Table 4:** Effectiveness of SB Interventions in Children Compared to an Active Control

Subgroup 1: Behavioural	Studies (***n***=)	I (***n***=)	C (***n***=)	I^**2**^	MD (95%CI)
Outcome 1: SB min/day (≤6mo)	4	87	90	0%	-48.26 [-69.83, -26.69]
Outcome 2: SB min/day (>6mo)	1	255	176	N/A	-4.00 [-13.16, 5.16]
**Subgroup 3: Mixed**	**Studies (*n*=)**	**I (*n*=)**	**C (*n*=)**	**I**^**2**^	**MD (95%CI)**
Outcome 1: SB min/day (≤6mo)	4	46	44	91%	-74.73 [-152.36, 2.90]
Outcome 2: SB min/day (>6mo)	1	22	22	N/A	19.14 [-3.46, 41.74]
Outcome 4: SB % (>6mo)	1	39	37	N/A	3.17 [-7.60, 13.94]
**Overall Results**	**Studies (*n*=)**	**I (*n*=)**	**C (*n*=)**	**I**^**2**^	**MD (95%CI)**
Outcome 1: SB min/day (≤6mo)	8	133	134	86%	-59.90 [-102.16, -17.65]
Outcome 2: SB min/day (>6mo)	2	277	198	71%	5.17 [-17.01, 27.35]
Outcome 4: SB % (>6mo)	1	39	37	N/A	3.17 [-7.60, 13.94]

The findings from the analyses of the children’s studies demonstrated moderate reductions in daily SB with behavioural, environmental and mixed interventions that included an inactive control, in the short-term. Results for the reductions in percentage SB time were not very robust due to the scarcity of data. Furthermore, findings for behavioural and mixed in the medium-term were inconclusive due to the lack of studies included in this outcome. The results from the children studies demonstrated extreme heterogeneity in behavioural interventions at short-term and mixed interventions at medium-term across the inactive control comparator outcomes. Within the active control outcomes, extreme heterogeneity was demonstrated in mixed interventions at short-term. In a sensitivity analysis, studies including children aged less than five were removed as potentially their environments may differ. Mixed interventions in children when compared to inactive controls became non-significant in the short term (-23.13; 95%CI -66.47, 20.21; n=3678; I^2^ 37%).

### GRADE Assessment

The GRADE approach was used to assess the certainty in evidence across all outcomes. The overall GRADE quality rating and scoring for risk of bias, imprecision, inconsistency, indirectness, and publication bias are presented in Supplement D. Due to the high heterogeneity across the outcomes, the majority were ranked as having a very low to low certainty of evidence.

## Discussion

The findings of the review have shown that behavioural and environmental interventions in adults resulted in a significant short-term (≤6 months) reduction in SB and interventions using a mix of strategies resulted in a statistically significant reduction in SB. In both the short and medium-term. The heterogeneity of the results was high in all cases, reducing the confidence in the consistency of the findings, in addition, the GRADE assessment demonstrated very low certainty of evidence against the relevant outcomes. In children, SB interventions with short-term follow-up (≤6 months) resulted in a statistically significant difference in favour of the intervention. Overall, there was high heterogeneity and the GRADE certainty of evidence assessment was rated low. For both adult and children studies, the complexity assessment revealed no association between effect size and complexity of the intervention, suggesting that complexity has no impact on the effectiveness of interventions. It is important to recognise that the complexity assessment is not without limitations. The tool is designed to be applied in reviews including a group of very similar interventions. However, interventions included within the current review varied in terms of the active components, the degree of tailoring and the nature of the causal pathways between the intervention and outcome. In order to avoid poor reporting, interventions were grouped based on their type (i.e. behavioural, environmental or mixed), whether the intervention included an active or inactive control and the length of follow-up. These steps were taken in order to avoid misinterpretation of data and promote comparison between similar studies as the tool intends. One of the main benefits of using this tool is that it facilitates an in-depth, systematic assessment of the complexity of interventions, leading to an increased understanding of how complex interventions work [34].

### Adult Interventions

Our findings are supported by previous systematic reviews in adults [23, 27, 29, 30], demonstrating that the interventions had a positive effect on reducing SB. In line with the findings from Peachey *et al*. which included studies published up to July 2017 [27], the findings from this review demonstrated the greatest reduction in sitting time from environmental interventions. These findings are interesting considering that the global complexity scores in environmental interventions were considerably lower than that of behavioural and mixed interventions. This suggests that within certain contexts where there is more control (i.e. workplace settings), less complex interventions targeting environmental determinants may be required. This finding is supported by a previous review by Gardner and colleagues [24] focusing on behaviour change strategies used in SB reduction interventions among adults, where interventions based on environmental restructuring, persuasion, or education were the most promising in terms of reducing SB. They also highlighted the most promising behaviour change techniques were self-monitoring, problem solving, and restructuring the social or physical environment [24].

Many of the environmental interventions included in the current review were based in the workplace and mainly involved adding height-adjustable desks or standing tables. However, most provided few details to individuals on how to effectively use the new apparatus which could account for the lack of change in daily SB time. Additionally, individuals may have needed extra behavioural elements to sustain the change (e.g. education on the benefits of reducing sitting in the long-term or activity trackers to provide feedback on progress towards goals). A systematic review of interventions for reducing non-occupational SB in adults and older adults found that interventions could reduce leisure sitting time in adults in the medium-term (-30 min/day; 95% CI -58 to -2), and TV viewing in the short-term (-61 min/day; 95% CI -79 to -43) and medium-term (-11 min/day; 95% CI -20 to -2) [192]. Similarly, the current study found greater reductions in SB were evident at short-term compared to medium-term across all outcomes with the exception of mixed interventions including an active control. However, this outcome only considered one study with a small sample size (n=36).

In terms of determining whether these reductions in daily SB are clinically meaningful, Healy and colleagues have modelled the impact of reallocating two daily hours of sitting to standing or to stepping on cardio-metabolic risk factors in adults [193]. This two-hour reduction is in line with recommendations from Public Health England and the Active Working Community Interest Company for office-based workers [194]. They found significant associations with sitting-to-standing reallocations included small-to-medium reductions in fasting plasma glucose (2%), triglycerides (11%) and total/HDL-cholesterol ratio (6%) while sitting-to-stepping reallocations were significantly associated with medium reductions in body mass index (11%), waist circumference (7.5cm), triglycerides (14%) and two-hour plasma glucose (11%) [193]. Despite the comparatively smaller reductions in SB time found in our review, these changes in SB could still have a clinically meaningful impact on adult’s health as a 30-minute per day reduction in daily sitting time has been suggested as an effective target for observing long-term health benefits in another systematic review [27].

Although this review appears to demonstrate the potential utility of SB-reducing interventions in adults in the medium-term (i.e. >6 months), stronger evidence exists for SB-reducing interventions in the short-term (≤6 months). There is a need for more high-quality studies to fully consider how to effectively reduce daily SB time in the months and years after interventions have been implemented. Findings demonstrate that SB interventions are effective, with environmental interventions demonstrating a window for opportunity. A range of theoretical frameworks were implemented across the SB interventions including, social cognitive theory, self-determination theory and the theory of planned behaviour. A recent systematic review described the behaviour change strategies used within interventions that sought to reduce SB [195]. In support of the current study, authors concluded that interventions based on environmental restructuring, persuasion or education were most effective. In addition, self-monitoring, problem solving and restructuring the social or physical environment were particularly promising behaviour change techniques. Future studies should consider theory-driven SB interventions delivered in the work-place as they could result in benefits for both the individual and the employer, high-quality studies need to be conducted to confirm these effects.

### Children Interventions

A body of evidence recognises that SB in childhood may have significant consequences for poor health outcomes in adulthood [19], making the development of effective interventions an important public health concern [196]. The current review included 84 studies in children (mean age 2-19 years) of which 62% [52] were carried out in a school setting. All interventions with the exceptions of those that were mixed including an active control in the medium-term demonstrated reductions in SB. A recent systematic review of interventions to reduce SB in 0-5 year olds found overall mean reductions in SB of -18.91 min/day; 95% CI -33.31 to -4.51 [197]. This finding suggests that interventions of ≥6 months duration and those conducted in community settings were more effective. The findings within the current review demonstrated stronger evidence for effectiveness in the short-term, however due to a lack of studies included in the medium-term analysis it is difficult to compare findings between the follow-up periods, and hence make conclusions on the available evidence.

Studies of SB interventions targeting children are mainly carried out within a school setting due to ease of access to the target population, with a large proportion of their week days, during term time spent in school [198–200]. Furthermore, evidence supporting the effect of school-based interventions which target out-of-school activity (i.e. changes to screen time) or overall activity are varied with most finding no effect [198–200], and some showing that changes are possible where curriculum changes can be made [201]. In a systematic review of school-based interventions, Hegarty and colleagues [202] demonstrated that multicomponent interventions may be an effective method for reducing device-based measured SB in the short-to-medium term. In line with the current review, multi-component interventions were most promising in terms of effectively reducing SB in children. These findings would suggest that mixed interventions, incorporating both behavioural and environmental components, may increase the likelihood of reducing sitting time in children. A review of reviews examining interventions designed to reduce SB among children and adolescents was conducted in 2013 [203]. Authors found that all reviews concluded some level of effectiveness in reducing time spent in SB, with most presenting a small but significant reduction in sedentary time. Effective strategies identified from the analysis revealed that involving the family, incorporating behaviour change techniques and including environmental components all positively supported the intervention effect. Consequently, future research should consider non-school settings such as home or community settings or seek to change curricular activity and SB policy to illicit benefits.

## Conclusions

The primary aim of this review was to synthesise and evaluate the complexity and effectiveness of current SB interventional approaches to reduce sedentary time across the lifespan. The findings of this review demonstrated that interventions may lead to relevant reductions in daily sedentary time. However, the heterogeneity in reported outcomes, intervention components, and control arms (active versus inactive) prevented us from drawing firmer conclusions from the evidence provided. The complexity assessment also suggested that interventions may need to be more complex to address the multi-faceted nature of SB, but a higher complexity score is not necessarily associated with better outcomes in terms of sustained change.

## Supplementary information

**Additional file 1: Supplement A.** Search Strategy

**Additional file 2: Supplement B.** Characteristics of Included Studies

**Additional file 3: Supplement C.** Risk of Bias

**Additional file 4. Supplement D.** Meta-Analysis and GRADE Assessment

## Data Availability

All data generated or analysed during this study are included in this published article and its supplementary information files.

## Abbreviations

ATK: Andrew T. Kunzmann
GRADE: Grading of Recommendations, Assessment, Development and Evaluations
IIM: Ilona I. McMullan
JJW: Jason J. Wilson
KW: Katharina Wirth
LCP: Laura Coll-Planas
MAT: Mark A. Tully
MGG: Maria Giné-Garriga
MR: Marta Roqué
MRC: Medical Research Council
NEB: Nicole E. Blackburn
PA: Physical activity
PC: Paolo Caserotti
RCT: Randomised controlled trial
SB: Sedentary behaviour
SBA: Sergi Blancafort Alias
SD: Standard deviation

## References

[1] Tremblay MS, Aubert S, Barnes JD, Saunders TJ, Carson V, Latimer-Cheung AE, Chastin SFM, Altenburg TM, Chinapaw MJM, Terminology Consensus Project Participants SBRN (2017). Sedentary Behaviour Research Network (SBRN) Terminology Consensus Project process and outcome. Int J Behav Nutr Physical Act.

[2] Prince SA, Reed JL, McFetridge C, Tremblay MS, Reid RD (2017). Correlates of sedentary behaviour in adults: a systematic review. Obes Rev.

[3] Dogra S, Stathosokostas L. Sedentary behaviour and physical activity are independent predictors of successful aging in middle-aged and older adults. J Aging Res. 2012:190654. 10.1155/2012/190654 PMC3446656 22997579

[4] De Rezende LF, Lopes MR, Rey-Lopez JP, Matsudo VK, do Carmo Luiz O (2014). Sedentary behavior and health outcomes: an overview of systematic reviews. PLOS One.

[5] Ekeland U, Tarp J, Steene-Johannessen J, Hansen BH, Jefferis B, Fagerland MW, Whincup P, Diaz KM, Hooker SP, Chernofsky A, Larson MG, Spartano N, Vasan RS, Dohrn I-M, Hagstromer M, Edwardson C, Yates T, Shiroma E, Anderssen SA, Lee I-M (2019). Dose-response associations between accelerometry measured physical activity and sedentary time and all cause mortality: systematic review and harmonised meta-analysis. Br Med J.

[6] Stamatakis E, Gale J, Bauman A, Ekeland U, Hamer M, Ding D (2019). Sitting time, physical activity, and risk of mortality in adults. J Am Coll Cardiol.

[7] Van der Ploeg H, P. & Hillsdon, M. Is sedentary behaviour just physical inactivity by another name? Int J Behav Nutr Physical Act. 2017;14(142). 10.1186/s12966-017-0601-0 PMC5651642 29058587

[8] Wilmot EG, Edwardson CL, Achana FA, Davies MJ, Gorely T, Gray LJ, Khunti K, Yates T, Biddle SJ (2012). Sedentary time in adults and the association with diabetes, cardiovascular disease and death: systematic review and meta-analysis. Diabetologia.

[9] Biswas A, Oh PI, Faulkner GE, Bajaj RR, Silver MA, Mitchell MS, Alter DA (2015). Sedentary time and its association with risk for disease incidence, mortality, and hospitalization in adults. Ann Internal Med.

[10] Zhou Y, Zhao H, Peng C (2015). Association of sedentary behaviour with the rosk of breast cancer in women: update meta-analysis of observational studies. Ann Epidemiol.

[11] Ma P, Yao Y, Sun W, Dai S, Zhou C (2017). Daily sedentary time and its association with risk for colorectal cancer in adults: A dose-response meta-analysis of prospective cohort studies. Medicine (Baltimore).

[12] Heron, L., O’Neill, C., McAneney, H., Kee, F., Tully, MA. (2019) Direct health costs of sedentary behaviour in the UK. Journal of Epidemiology and Community Health, Published Online First: 25 March 2019. doi: 10.1136/jech-2018-211758. 10.1136/jech-2018-211758 30910857

[13] World Health Organisation (2011) Global Recommendations on Physical Activity for Health. Accessed on: https://www.who.int/dietphysicalactivity/physical-activity-recommendations-18-64years.pdf. 26180873

[14] Department of Health (DoH) (2019) UK Chief Medical Officers' Physical Activity Guidleines. Accessed on 12 September 2019. Available at: https://assets.publishing.service.gov.uk/government/uploads/system/uploads/attachment_data/file/832868/uk-chief-medical-officers-physical-activity-guidelines.pdf.

[15] Owens N, Sugiyama T, Eakin EE, Gardiner PA, Tremblay MS, Sallis JF (2011). Adults’ Sedentary Behvaiour Determinants and Interventions. Am J Prevent Med.

[16] Hadgraft NT, Lynch BM, Clark BK, Healy GN, Owen N, Dunstan DW. Excessive sitting at work and at home: Correlates of occupational sitting and TV viewing time in working adults. BMC Public Health. 2015;15(899). 10.1186/s12889-015-2243-y PMC4571074 26374514

[17] Bernaards CM, Hildebrandt VH, Hendriksen IJ. Correlates of sedentary time in different age groups: results from a large cross sectional Dutch survey. BMC Public Health. 2016;16(1121).. 10.1186/s12889-016-3769-3 PMC5080694 27784297

[18] Thorp AA, Owen N, Neuhaus M, Dunstan DW (2011). Sedentary Behaviours and Subsequent Health Outcomes in Adults: A Systematic Review of Longitudinal Studies, 1996-2011. Am J Prevent Med.

[19] de Rezende LF, Rey-López JP, Matsudo VK, do Carmo Luiz O (2014). Sedentary behaviour and health outcomes among older adults: a systematic review. BMC Public Health.

[20] Carson V, Hunter S, Kuzik N, Gray CE, Poitras VJ, Chaput J-P, Saunders TJ, Katzmarzyk PT, Okely AD, Gorber SC, Kho ME, Sampson M, Lee H, Tremblay MS (2016). Systematic review of sedentary behaviour and health indicators in school-aged children and youth: an update. Appl Physiol Nutr Metab.

[21] Manini TM, Carr LJ, King AC, Marshall S, Robinson TN, Rejeski WJ (2015). Interventions to Reduce Sedentary Behaviour. Med Sci Sports Exerc.

[22] Hinckson E, Salmon J, Benden M, Clemes SA, Sudholz B, Barber SE, Aminian S, Ridgers ND (2016). Standing Classrooms: Research and Lessons Learned from Around the World. Sports Med.

[23] Shrestha, N., Kukkonen-Harjula, KT, Verbeek, JH., Ijaz, S., Hermans, V. & Bhaumik, S. (2018) Workplace interventions for reducing sitting at work. Cochrane Database Syst Rev, (6) DOI: 10.1002/14651858.CD010912.pub4. 10.1002/14651858.CD010912.pub4 PMC6513236 29926475

[24] Gardner B, Smith L, Lorencatto F, Hamer M, Biddle S, J, H. How to reduce sitting time? A review of behaviour change strategies used in sedentary behaviour reduction interventions among adults. Health Psychol Rev J. 2016;10(1). 10.1080/17437199.2015.1082146 PMC4743603 26315814

[25] Michie S, West R, Sheals K, Godinho C, A. (2018). Evaluating the effectiveness of behavior change techniques in health-related behavior: a scoping review of methods used.Translational. Behav Med.

[26] Coldrey M. Approaches to Changing Behaviours: Designing an Intervention to Reduce Sedentary Behaviour in the Workplace using Behaviour Change Theory. J Phys Fitness Med Treat Sports. 2018;4(2).

[27] Peachey, M.M., Richardson, J., Tang, AV, Dal-Bello Haas, V. & Gravesande J. (2018) Environmental, behavioural and multicomponent interventions to reduce adults' sitting time: a systematic review and meta-analysis. Br J Sports Meddoi: 10.1136/bjsports-2017-098968. [Epub ahead of print]. 10.1136/bjsports-2017-098968 30352864

[28] O'Cathain A, Croot L, Duncan E (2019). Guidance on how to develop complex interventions to improve health and healthcare. BMJ Open.

[29] Martin, A., Fitzsimons, C., Jepson, R., Saunders, DH., van der Ploeg, HP., Teixeira, PJ., Gray, CM, & Mutrie, N.; EuroFIT consortium (2015) Interventions with potential to reduce sedentary time in adults: systematic review and meta-analysis. Br J Sports Med, 49(16), 1056-1063. 10.1136/bjsports-2014-094524 25907181

[30] Prince SA, Saunders TJ, Gresty K, Reid RD (2014). A comparison of the effectiveness of physical activity and sedentary behaviour interventions in reducing sedentary time in adults: a systematic review and meta-analysis of controlled trials. Obes Rev.

[31] Moher, D., Liberati, A., Tetzlaff, J., Altman, DG, & The PRISMA Group (2009) Preferred reporting items for systematic reviews and meta-analyses: the PRISMA statement. PLOS Med, 6(7). PMC3090117 21603045

[32] Higgins, J, P, T., Chandler, J., Cumpston, M., Li, T., Page, M,J. & Welch, V,A. (2019) Cochrane handbook for systematic reviews of interventions version 6.0: The Cochrane Collaboration. Accessed on: http://handbook.cochrane.org.

[33] Guyatt GH, Oxman AD, Kunz R, Vist GE, Falck-Ytter Y, Schunemann HJ (2008). What is "quality of evidence" and why is it important to clinicians?. Br Med J.

[34] Lewin S, Hendry M, Candler J, Ocman AD, Michie S, Shepperd S, Reeves BC, Tugwell P, Hannes K, Rehfuess EA, Welch V, McKenzie JE, Burford B, Petkovic J, Anderson LM, Harris J, Noyes J. Assessing the complexity of interventions within systematic reviews: development, content and use of a new tool (iCAT_SR). BMC Med Res Methodol. 2017;17(76). 10.1186/s12874-017-0349-x PMC5406941 28446138

[35] Aadahl M, Linneberg A, Møller T, Rosenørn S, Dunstan D, Witte D, Jørgensen T (2014). Motivational counseling to reduce sitting time: A community-based randomized controlled trial in adults. Am J Prevent Med.

[36] Adams M, Davis P, Gill D. A hybrid online intervention for reducing sedentary behavior in obese women. Front Public Health. 2013;1(45). 10.3389/fpubh.2013.00045 PMC3859962 24350214

[37] Aguiñaga S, Marquez D (2019). Impact of Latin Dance on Physical Activity, Cardiorespiratory fitness, and sedentary behavior among Latinos attending an adult day center. J Aging Health.

[38] Alkhajah T, Reeves M, Eakin E, Winkler E, Owen N, Healy G (2012). Sit-stand workstations: A pilot intervention to reduce office sitting time. Am J Prevent Med.

[39] Arrogi A, Schotte A, Bogaerts A, Boen F, Seghers J. Short- and long-term effectiveness of a three-month individualized need-supportive physical activity counseling intervention at the workplace. BMC Public Health. 2017;17(52). 10.1186/s12889-016-3965-1 PMC5223544 28069016

[40] Arrogi A, Bogaerts A, Seghers J, Devloo K, Vanden Abeele V, Geurts L (2017). Evaluation of stAPP: A smartphone-based intervention to reduce prolonged sitting among Belgian adults. Health Promot Int.

[41] Balducci S, D'Errico V, Haxhi J, Sacchetti M, Orlando G, Cardelli P, Pugliese G (2019). Effect of a Behavioral Intervention Strategy on Sustained Change in Physical Activity and Sedentary Behavior in Patients with Type 2 Diabetes: The IDES-2 Randomized Clinical Trial. JAMA.

[42] Barbieri D, Srinivasan D, Mathiassen S, Oliveira A (2017). Comparison of Sedentary Behaviors in Office Workers Using Sit-Stand Tables with and Without Semiautomated Position Changes. Human Factors.

[43] Barone Gibbs B, Brach J, Byard T, Creasy S, Davis K, McCoy S, Jakicic J (2017). Reducing Sedentary Behavior Versus Increasing Moderate-to-Vigorous Intensity Physical Activity in Older Adults: A 12-Week Randomized, Clinical Trial. J Aging Health.

[44] Barone Gibbs, B., Hergenroeder, A., Perdomo, S., Kowalsky, R., Delitto, A., & Jakicic, J. (2018) Reducing sedentary behaviour to decrease chronic low back pain: the stand back randomised trial. Occupational and Environmental Medicine, oemed-2017-104732. 10.1136/oemed-2017-104732 PMC8283944 29330230

[45] Barwais F, Cuddihy T, Tomson L. Physical activity, sedentary behavior and total wellness changes among sedentary adults: A 4-week randomized controlled trial. Health Qual Life Outcomes. 2013;11(1). 10.1186/1477-7525-11-183 PMC4228472 24168638

[46] Biddle, S., Edwardson, C., Wilmot, E., Yates, T., Gorely, T., Bodicoat, D., . . . Davies, M. (2015) A randomised controlled trial to reduce sedentary time in young adults at risk of type 2 diabetes mellitus: Project STAND (Sedentary Time and Diabetes) PLoS ONE, 10(12). 10.1371/journal.pone.0143398 PMC4666612 26623654

[47] Carr L, Leonhard C, Tucker S, Fethke N, Benzo R, Gerr F (2016). Total worker health intervention increases activity of sedentary workers. Am J Preventive Med.

[48] Chang AK, Fritschi C, Kim MJ (2013). Sedentary behavior, physical activity, and psychological health of Korean older adults with hypertension: effect of an empowerment intervention. Res Gerontol Nurs.

[49] Chau J, Daley M, Dunn S, Srinivasan A, Do A, Bauman A, van der Ploeg H. The effectiveness of sit-stand workstations for changing office workers' sitting time: Results from the Stand@Work randomized controlled trial pilot. Int J Behav Nutr Physical Act. 2014;11(1). 10.1186/s12966-014-0127-7 PMC4194364 25291960

[50] Chau J, Sukala W, Fedel K, Do A, Engelen L, Kingham M, Bauman A (2016). More standing and just as productive: Effects of a sit-stand desk intervention on call center workers' sitting, standing, and productivity at work in the Opt to Stand pilot study. Preventive Med Rep.

[51] Chiang L, Heitkemper M, Chiang S, Tzeng W, Lee M, Hung Y, Lin C (2019). Motivational counseling to reduce sedentary behaviors and depressive symptoms and improve health-related quality of life among women with metabolic syndrome. J Cardiovasc Nurs.

[52] Danquah I, Kloster S, Holtermann A, Aadahl M, Bauman A, Ersbøll A, Tolstrup J (2017). Take a Stand!-A multi-component intervention aimed at reducing sitting time among office workers-a cluster randomized trial. Int J Epidemiol.

[53] De Cocker K, De Bourdeaudhuij I, Cardon G, Vandelanotte C. The effectiveness of a web-based computer-tailored intervention on workplace sitting: A randomized controlled trial. J Med Int Res. 2016;18(5). 10.2196/jmir.5266 PMC4908304 27245789

[54] De Greef K, Deforche B, Tudor-Locke C, De Bourdeaudhuij I (2010). A cognitive-behavioural pedometer-based group intervention on physical activity and sedentary behaviour in individuals with type 2 diabetes. Health Educ Res.

[55] De Greef K, Deforche B, Ruige J, Bouckaert J, Tudor-Locke C, Kaufman J, De Bourdeaudhuij I (2011). The effects of a pedometer-based behavioral modification program with telephone support on physical activity and sedentary behavior in type 2 diabetes patients. Patient Educ Counseling.

[56] Donath L, Faude O, Schefer Y, Roth R, Zahner L (2015). Repetitive daily point of choice prompts and occupational sit-stand transfers, concentration and neuromuscular performance in office workers: An RCT. Int J Environ Res Public Health.

[57] Dutta N, Koepp G, Stovitz S, Levine J, Pereira M (2014). Using sit-stand workstations to decrease sedentary time in office workers: A randomized crossover trial. Int J Environ Res Public Health.

[58] Edwardson, C., Yates, T., Biddle, S., Davies, M., Dunstan, D., Esliger, D., . . . Munir, F. (2018) Effectiveness of the stand more at (SMArT) work intervention: Cluster randomised controlled trial. BMJ (Online), 363. 10.1136/bmj.k3870 PMC6174726 30305278

[59] English C, Healy GN, Olds T, Parfitt G, Borkoles E, Coates A, Kramer S, Bernhardt J (2016). Reducing sitting time after stroke: A Phase II safety and feasibility randomized controlled trial. Arch Physical Med Rehabil.

[60] Evans R, Fawole H, Sheriff S, Dall P, Grant P, Ryan C (2012). Point-of-choice prompts to reduce sitting time at work: A randomized trial. Am J Preventive Med.

[61] Fanning J, Porter G, Awick E, Wójcicki T, Gothe N, Roberts S, McAuley E (2016). Effects of a DVD-delivered exercise program on patterns of sedentary behavior in older adults: A randomized controlled trial. Preventive Med Rep.

[62] Frank L, Hong A, Ngo V (2019). Causal evaluation of urban greenway retrofit: A longitudinal study on physical activity and sedentary behavior. Preventive Med.

[63] Gao Y, Nevala N, Cronin N, Finni T (2016). Effects of environmental intervention on sedentary time, musculoskeletal comfort and work ability in office workers. Eur J Sport Sci.

[64] Gentile D, Welk G, Eisenmann J, Reimer R, Walsh D, Russell D, Fritz K (2009). Evaluation of a multiple ecological level child obesity prevention program: Switch® what you Do, View, and Chew. BMC Med.

[65] Gilson, N., Puig-Ribera, A., McKenna, J., Brown, W., Burton, N., & Cooke, C. (2009) Do walking strategies to increase physical activity reduce reported sitting in workplaces: A randomized control trial. International Journal of Behavioral Nutrition and Physical Activity, 6. 10.1186/1479-5868-6-43 PMC2717045 19619295

[66] Graves L, Murphy R, Shepherd S, Cabot J, Hopkins N. Evaluation of sit-stand workstations in an office setting: A randomised controlled trial. BMC Public Health. 2015;15(1). 10.1186/s12889-015-2469-8 PMC4653846 26584856

[67] Hallman D, Mathiassen S, Jahncke H (2018). Sitting patterns after relocation to activity-based offices: A controlled study of a natural intervention. Preventive Med.

[68] Haslam C, Kazi A, Duncan M, Clemes S, Twumasi R (2019). Walking Works Wonders: a tailored workplace intervention evaluated over 24 months. Ergonomics.

[69] Healy G, Eakin E, Owen N, LaMontagne A, Moodie M, Winkler E, Dunstan D (2016). A cluster RCT to reduce office workers’ sitting time: Impact on activity outcomes. Med Sci Sports Exerc.

[70] Jago R, Sebire S, Turner K, Bentley G, Goodred J, Fox K, Lucas P. Feasibility trial evaluation of a physical activity and screen-viewing course for parents of 6 to 8 year-old children: Teamplay. Int J Behav Nutr Physical Act. 2013;10(31). 10.1186/1479-5868-10-31 PMC3598924 23510646

[71] Júdice P, Hamilton M, Sardinha L, Silva A. Randomized controlled pilot of an intervention to reduce and break-up overweight/obese adults' overall sitting-time. Trials. 2015;16(1). 10.1186/s13063-015-1015-4 PMC4631103 26525049

[72] Kerr J, Takemoto M, Bolling K, Atkin A, Carlson J, Rosenberg D, Merchant G. Two-arm randomized pilot intervention trial to decrease sitting time and increase sit-to-stand transitions in working and non-working older adults. PLoS One. 2016;11(1). 10.1371/journal.pone.0145427 PMC4703201 26735919

[73] Klaren R, Hubbard E, Motl R (2014). Efficacy of a behavioral intervention for reducing sedentary behavior in persons with multiple sclerosis: A pilot examination. Am J Preventive Med.

[74] Knowlden A, Sharma M (2016). One-year efficacy testing of enabling mothers to prevent pediatric obesity through web-based education and reciprocal determinism (EMPOWER) randomized control trial. Health Educ Behav.

[75] Kozey-Keadle S, Staudenmayer J, Libertine A, Mavilia M, Lyden K, Braun B, Freedson P (2015). Changes in sedentary time and physical activity in response to an exercise training and/or lifestyle intervention. J Physical Act Health.

[76] Larouche M, Mullane S, Toledo M, Pereira M, Huberty J, Ainsworth B, Buman M. Using point-of-choice prompts to reduce sedentary behavior in sit-stand workstation users. Front Public Health. 2018;6. 10.3389/fpubh.2018.00323 PMC6258737 30525017

[77] Li I, Mackey M, Foley B, Pappas E, Edwards K, Chau J, Stamatakis E (2017). Reducing Office Workers’ Sitting Time at Work Using Sit-Stand Protocols. J Occupational Environ Med.

[78] Lin Y, Hong O, Lin C, Lu S, Chen M, Lee K (2018). A "Sit Less, Walk More" workplace intervention for office workers. J Occupational Environ Med.

[79] Lynch B, Nguyen N, Moore M, Reeves M, Rosenberg D, Boyle T, et al. A randomized controlled trial of a wearable technology-based intervention for increasing moderate to vigorous physical activity and reducing sedentary behavior in breast cancer survivors: The ACTIVATE Trial. Cancer. 2019. 10.1002/cncr.32143 31012970

[80] MacEwen B, Saunders T, MacDonald DJ, Burr JF (2017). Sit-stand desks to reduce workplace sitting time In office workers with abdominal obesity: A randomized controlled trial. J Physical Act Health.

[81] Maher J, Sliwinski M, Conroy D (2017). Feasibility and preliminary efficacy of an intervention to reduce older adults’ sedentary behavior. Transl Behav Med.

[82] Mitchell B, Smith A, Rowlands A, Fraysse F, Parfitt G, Lewis N, Dollman J (2019). Promoting physical activity in rural Australian adults using an online intervention. J Sci Med Sport.

[83] Neuhaus M, Healy G, Dunstan D, Owen N, Eakin E (2014). Workplace sitting and height-adjustable workstations: A randomized controlled trial. Am J Preventive Med.

[84] O'Dolan C, Grant M, Lawrence M, Dall P. A randomised feasibility study to investigate the impact of education and the addition of prompts on the sedentary behaviour of office workers. Pilot Feasibility Stud. 2018;4(1). 10.1186/s40814-017-0226-8 PMC5769488 29372071

[85] Orme M, Weedon A, Saukko P, Esliger D, Morgan M, Steiner M, Singh S. Findings of the chronic obstructive pulmonary disease-sitting and exacerbations trial (COPD-SEAT) in reducing sedentary time using wearable and mobile technologies with educational support: Randomized controlled feasibility trial. J Med Int Res. 2018;20(4). 10.2196/mhealth.9398 PMC5917078 29643055

[86] Overgaard K, Nannerup K, Lunen M, Maindal H, Larsen R (2018). Exercise more or sit less? A randomized trial assessing the feasibility of two advice-based interventions in obese inactive adults. J Sci Med Sport.

[87] Parry S, Straker L, Gilson N, Smith A. Participatory workplace interventions can reduce sedentary time for office workers - A randomised controlled trial. PLoS One. 2013;8(11). 10.1371/journal.pone.0078957 PMC3827087 24265734

[88] Pedersen S, Cooley P, Mainsbridge C (2014). An e-health intervention designed to increase workday energy expenditure by reducing prolonged occupational sitting habits. Work.

[89] Prince S, Reed J, Cotie L, Harris J, Pipe A, Reid R (2018). Results of the Sedentary Intervention Trial in Cardiac Rehabilitation (SIT-CR Study): A pilot randomized controlled trial. Int J Cardiol.

[90] Pronk N, Katz A, Lowry M, Payfer J. Reducing Occupational Sitting Time and Improving Worker Health: The Take-a-Stand Project, 2011. Preventing Chronic Dis. 2012;9. 10.5888/pcd9.110323 PMC3477898 23057991

[91] Puig-Ribera A, Bort-Roig J, González-Suárez A, Martínez-Lemos I, Giné-Garriga M, Forto J, et al. Patterns of impact resulting from a 'sit less, move more' web-based program in sedentary office employees. PLoS One. 2015;10(4). 10.1371/journal.pone.0122474 PMC4382156 25830782

[92] Raynor H, Steeves E, Bassett D, Thompson D, Gorin A, Bond D (2013). Reducing TV watching during adult obesity treatment: Two pilot randomized controlled trials. Behavior Ther.

[93] Schuna J, Swift D, Hendrick C, Duet M, Johnson W, Martin C, Tudor-Locke C (2014). Evaluation of a workplace treadmill desk intervention. J Occupational Environ Med.

[94] Spring B, Schneider K, McFadden H, Vaughn J, Kozak A, Smith M, Lloyd-Jones D (2012). Multiple behavior changes in diet and activity: A randomized controlled trial using mobile technology. Arch Internal Med.

[95] Spring B, Pellegrini C, McFadden H, Pfammatter A, Stump T, Siddique J, Hedeker D. Multicomponent mHealth intervention for large, sustained change in multiple diet and activity risk behaviors: The make better choices 2 randomized controlled trial. J Med Internet Res. 2018;20(6). 10.2196/10528 PMC6030572 29921561

[96] Steeves, J., Bassett, D., Fitzhugh, E., Raynor, H., & Thompson, D. (2012) Can sedentary behavior be made more active? A randomized pilot study of TV commercial stepping versus walking. International Journal of Behavioral Nutrition and Physical Activity, 9. 10.1186/1479-5868-9-95 PMC3487755 22866941

[97] Stephens S, Winkler E, Trost S, Dunstan D, Eakin E, Chastin S, Healy G (2014). Intervening to reduce workplace sitting time: How and when do changes to sitting time occur?. Br J Sports Med.

[98] Sui W, Prapavessis H (2018). Standing Up for Student Health: An application of the health action process approach for reducing student sedentary behavior—randomised control pilot trial. Appl Psychol.

[99] Schwartz B, Kapellusch JM, Baca A, Wessner B (2019). Medium-term effects of a two-desk sit/stand workstation on cognitive performance and workload for healthy people performing sedentary work: a secondary analysis of a randomised controlled trial. Ergonomics.

[100] Taylor, W., Paxton, R., Shegog, R., Coan, S., Dubin, A., Page, T., & Rempel, D. (2016) Impact of Booster Breaks and Computer Prompts on Physical Activity and Sedentary Behavior Among Desk-Based Workers: A Cluster-Randomized Controlled Trial. Preventing Chronic Disease, 13. 10.5888/pcd13.160231 PMC5127177 27854422

[101] Ter Hoeve N, Sunamura M, Stam H, Boersma E, Geleijnse M, van Domburg R, van den Berg-Emons R (2018). Effects of two behavioral cardiac rehabilitation interventions on physical activity: A randomized controlled trial. Int J Cardiol.

[102] Thomsen T, Aadahl M, Beyer N, Hetland M, Løppenthin K, Midtgaard J, et al. Motivational counselling and SMS-reminders for reduction of daily sitting time in patients with rheumatoid arthritis: A descriptive randomised controlled feasibility study. BMC Musculoskeletal Disord. 2016;17(1). 10.1186/s12891-016-1266-6 PMC5070122 27756265

[103] Thomsen T, Aadahl M, Beyer N, Hetland M, Løppenthin K, Midtgaard J (2017). The efficacy of motivational counselling and SMS reminders on daily sitting time in patients with rheumatoid arthritis: A randomised controlled trial. Ann Rheumatic Dis.

[104] Tobin R, Leavy J, Jancey J (2016). Uprising: An examination of sit-stand workstations, mental health and work ability in sedentary office workers, in Western Australia. Work.

[105] Tuominen P, Husu P, Raitanen J, Kujala U, Luoto R. The effect of a movement-to-music video program on the objectively measured sedentary time and physical activity of preschool-aged children and their mothers: A randomized controlled trial. PLoS One. 2017;12(8). 10.1371/journal.pone.0183317 PMC5578653 28859091

[106] Urda J, Lynn J, Gorman A, Larouere B (2016). Effects of a Minimal Workplace Intervention to Reduce Sedentary Behaviors and Improve Perceived Wellness in Middle-Aged Women Office Workers. J Physical Act Health.

[107] Verweij L, Proper K, Weel A, Hulshof C, Van Mechelen W (2012). The application of an occupational health guideline reduces sedentary behaviour and increases fruit intake at work: Results from an RCT. Occupational Environ Med.

[108] Whaley S, McGregor S, Jiang L, Gomez J, Harrison G, Jenks E. A WIC-based intervention to prevent early childhood overweight. J Nutr Educ Behav. 2010;42(3). 10.1016/j.jneb.2010.02.010 20399409

[109] Wyke S, Bunn C, Andersen E, Silva M, van Nassau F, McSkimming P, van der Ploeg H. The effect of a programme to improve men’s sedentary time and physical activity: The european fans in training (EuroFIT) randomised controlled trial. PLoS Med. 2019;16(2). 10.1371/journal.pmed.1002736 PMC6363143 30721231

[110] Zhu W, Gutierrez M, Toledo M, Mullane S, Stella A, Diemar R (2018). Long-term effects of sit-stand workstations on workplace sitting: A natural experiment. J Sci Med Sport.

[111] Aittasalo M, Jussila A, Tokola K, Sievänen H, Vähä-Ypyä H, Vasankari T. Kids Out; Evaluation of a brief multimodal cluster randomized intervention integrated in health education lessons to increase physical activity and reduce sedentary behavior among eighth graders. BMC Public Health. 2019;19(1). 10.1186/s12889-019-6737-x PMC6472104 30995905

[112] Andrade S, Lachat C, Ochoa-Aviles A, Verstraeten R, Huybregts L, Roberfroid D, Kolsteren P. A school-based intervention improves physical fitness in Ecuadorian adolescents: A cluster-randomized controlled trial. Int J Behav Nutr Physical Act. 2014;11(1). 10.1186/s12966-014-0153-5 PMC4272792 25490946

[113] Andrade S, Verloigne M, Cardon G, Kolsteren P, Ochoa-Avilés A, Verstraeten R, et al. School-based intervention on healthy behaviour among Ecuadorian adolescents: Effect of a cluster-randomized controlled trial on screen-time Health behavior, health promotion and society. BMC Public Health. 2015;15(1). 10.1186/s12889-015-2274-4 PMC4580309 26395439

[114] Ayala A, Salmon J, Timperio A, Sudholz B, Ridgers N, Sethi P, Dunstan D. Impact of an 8-month trial using height-adjustable desks on children’s classroom sitting patterns and markers of cardio-metabolic and musculoskeletal health. Int J Environ Res Public Health. 2016;13(12). 10.3390/ijerph13121227 PMC5201368 27973414

[115] Babic M, Smith J, Morgan P, Lonsdale C, Plotnikoff R, Eather N (2016). Intervention to reduce recreational screen-time in adolescents: Outcomes and mediators from the ‘Switch-Off 4 Healthy Minds’ (S4HM) cluster randomized controlled trial. Preventive Med.

[116] Bergh I, Van Stralen M, Bjelland M, Grydeland M, Lien N, Klepp K, et al. Post-intervention effects on screen behaviours and mediating effect of parental regulation: The Health in Adolescents study - A multi-component school-based randomized controlled trial. BMC Public Health. 2014;14(1). 10.1186/1471-2458-14-200 PMC3946033 24568125

[117] Bickham D, Hswen Y, Slaby R, Rich M (2018). A preliminary evaluation of a school-based media education and reduction intervention. J Prim Prevent.

[118] Birken C, Maguire J, Mekky M, Manlhiot C, Beck C, DeGroot J, Parkin P (2012). Office-based randomized controlled trial to reduce screen time in preschool children. Pediatrics.

[119] Bjelland, M., Bergh, I., Grydeland, M., Klepp, K., Andersen, L., Anderssen, S., Lien, N. (2011) Changes in adolescents' intake of sugar-sweetened beverages and sedentary behaviour: Results at 8 month mid-way assessment of the HEIA study - a comprehensive, multi-component school-based randomized trial. International Journal of Behavioral Nutrition and Physical Activity, 8. 10.1186/1479-5868-8-63 PMC3141615 21679476

[120] Brittin J, Frerichs L, Sirard J, Wells N, Myers B, Garcia J, Huang T. Impacts of active school design on school-time sedentary behavior and physical activity: A pilot natural experiment. PLoS One. 2017;12(12). 10.1371/journal.pone.0189236 PMC5720751 29216300

[121] Byun W, Lau E, Brusseau T. Feasibility and effectiveness of a wearable technology-based physical activity intervention in preschoolers: A pilot study. Int J Environ Res Public Health. 2018;15(9). 10.3390/ijerph15091821 PMC6163401 30142911

[122] Carson, V., Salmon, J., Arundell, L., Ridgers, N., Cerin, E., Brown, H., Crawford, D. (2013) Examination of mid-intervention mediating effects on objectively assessed sedentary time among children in the Transform-Us! cluster-randomized controlled trial. International Journal of Behavioral Nutrition and Physical Activity, 10. 10.1186/1479-5868-10-62 PMC3681598 23688180

[123] Cespedes E, Horan C, Gillman M, Gortmaker S, Price S, Rifas-Shiman S (2014). Participant characteristics and intervention processes associated with reductions in television viewing in the High Five for Kids study. Preventive Med.

[124] Chesham R, Booth J, Sweeney E, Ryde G, Gorely T, Brooks N, Moran C (2018). The Daily Mile makes primary school children more active, less sedentary and improves their fitness and body composition: a quasi-experimental pilot study. BMC Medicine.

[125] Chin A Paw, MJ., Singh, A., Brug, J., & van Mechelen, W. (2008) Why did soft drink consumption decrease but screen time not? Mediating mechanisms in a school-based obesity prevention program. International Journal of Behavioral Nutrition and Physical Activity, 5. 10.1186/1479-5868-5-41 PMC2542394 18694483

[126] Clemes S, Barber S, Bingham D, Ridgers N, Fletcher E, Pearson N, Dunstan D (2016). Reducing children's classroom sitting time using sit-to-stand desks: Findings from pilot studies in UK and Australian primary schools. J Public Health (United Kingdom).

[127] D'Haese S, Van Dyck D, De Bourdeaudhuij I, Deforche B, Cardon G. Organizing "Play Streets" during school vacations can increase physical activity and decrease sedentary time in children. Int J Behav Nutr Physical Act. 2015;12(1). 10.1186/s12966-015-0171-y PMC4334854 25888734

[128] de Bourdeaudhuij I, Verbestel V, De Henauw S, Maes L, Huybrechts I, Mårild S, Pigeot I (2015). Behavioural effects of a community-oriented setting-based intervention for prevention of childhood obesity in eight European countries. Main results from the IDEFICS study. Obes Rev.

[129] De Craemer M, De Decker E, Verloigne M, De Bourdeaudhuij I, Manios Y, Cardon G. The effect of a cluster randomised control trial on objectively measured sedentary time and parental reports of time spent in sedentary activities in Belgian preschoolers: The ToyBox-study. Int J Behav Nutr Physical Act. 2016;13(1). 10.1186/s12966-015-0325-y PMC4702324 26733186

[130] De Lepeleere S, De Bourdeaudhuij I, Cardon G, Verloigne M. The effect of an online video intervention ‘Movie Models’ on specific parenting practices and parental self-efficacy related to children’s physical activity, screen-time and healthy diet: a quasi experimental study. BMC Public Health. 2017;17(1). 10.1186/s12889-017-4264-1 PMC5408449 28449658

[131] Dennison B, Russo T, Burdick P, Jenkins P (2004). An intervention to reduce television viewing by preschool children. Arch Pediatr Adolesc Med.

[132] Downing K, Salmon J, Hinkley T, Hnatiuk J, Hesketh K. Feasibility and efficacy of a parent-focused, text message-delivered intervention to reduce sedentary behavior in 2- to 4-year-old children (mini movers): Pilot randomized controlled trial. J Med Internet Res. 2018;20(2). 10.2196/mhealth.8573 PMC5889816 29426816

[133] Ee J, Parry S, de Oliveira B, McVeigh J, Howie E, Straker L. Does a classroom standing desk intervention modify standing and sitting behaviour and musculoskeletal symptoms during school time and physical activity during waking time? Int J Environ Res Public Health. 2018;15(8). 10.3390/ijerph15081668 PMC6121556 30082657

[134] Ellis Y, Cliff D, Howard S, Okely A (2019). Feasibility, acceptability, and potential efficacy of a childcare-based intervention to reduce sitting time among pre-schoolers: A pilot randomised controlled trial. J Sports Sci.

[135] Epstein L, Paluch R, Constance M, Gordy C, Dorn J (2000). Decreasing Sedentary Behaviors in Treating Pediatric Obesity. Arch Pediatr Adolesc Med.

[136] Epstein L, Paluch R, Kilanowski C, Raynor H (2004). The effect of reinforcement or stimulus control to reduce sedentary behavior in the treatment of pediatric obesity. Health Psychology.

[137] Epstein L, Roemmich J, Robinson J, Paluch R, Winiewicz D, Fuerch J, Robinson M (2008). A Randomized Trial of the Effects of Reducing Television Viewing and Computer Use on Body Mass Index in Young Children. Arch Pediatr Adolesc Med.

[138] Escobar-Chaves L, Markham C, Addy R, Greisinger A, Murray N, Brehm B (2010). The fun families study: Intervention to reduce childrens TV viewing. Obesity.

[139] Faith M, Berman N, Heo M, Pietrobelli A, Gallagher D, Epstein L, Allison D (2004). Effects of contingent television on physical activity and television viewing in obese children. Pediatrics.

[140] Farley T, Meriwether R, Baker E, Watkins L, Johnson C, Webber L (2007). Safe play spaces to promote physical activity in inner-city children: Results from a pilot study of an environmental intervention. Am J Public Health.

[141] Fassnacht D, Ali K, Silva C, Gonçalves S, Machado P (2014). Use of text messaging services to promote health behaviors in children. J Nutr Educ Behav.

[142] Fitzgibbon M, Stolley M, Schiffer L, Van Horn L, Kauferchristoffel K, Dyer A (2005). Two-year follow-up results for Hip-Hop to Health Jr.: A randomized controlled trial for overweight prevention in preschool minority children. J Pediatrics.

[143] Fitzgibbon M, Stolley M, Schiffer L, Braunschweig C, Gomez S, Van Horn L, Dyer A (2011). Hip-hop to health Jr. Obesity prevention effectiveness trial: Postintervention results. Obesity.

[144] Ford B, Mcdonald T, Owens A, Robinson T (2002). Primary care interventions to reduce television viewing in African-American children. Am J Preventive Med.

[145] Foster G, Sherman S, Borradaile K, Grundy K, Vander Veur S, Nachmani J, Shults J (2008). A policy-based school intervention to prevent overweight and obesity. Pediatrics.

[146] French S, Sherwood N, JaKa M, Haapala J, Ebbeling C, Ludwig D (2016). Physical changes in the home environment to reduce television viewing and sugar-sweetened beverage consumption among 5- to 12-year-old children: a randomized pilot study. Pediatric Obesity.

[147] Goldfield G, Mallory R, Parker T, Cunningham T, Legg C, Lumb A (2006). Effects of open-loop feedback on physical activity and television viewing in overweight and obese children: A randomized, controlled trial. Pediatrics.

[148] Gortmaker S, Peterson K, Wiecha J, Sobol AM, Dixit S, Fox MK, Laird N (1999). Reducing obesity via a school-based interdisciplinary intervention among youth: Planet Health. Arch Pediatr Adolesc Med.

[149] Gortmaker S, Sobol A, Cheung L, Peterson K, Chomitz G, Cradle J, Bullock R (1999). Impact of a school-based interdisciplinary intervention on diet and physical activity among urban primary school children: Eat well and keep moving. Arch Pediatr Adolesc Med.

[150] Haines J, McDonald J, O'Brien A, Sherry B, Bottino C, Schmidt M, Taveras E (2013). Healthy habits, happy homes: Randomized trial to improve household routines for obesity prevention among preschool-aged children. JAMA Pediatrics.

[151] Harrison M, Burns C, McGuinness M, Heslin J, Murphy N (2006). Influence of a health education intervention on physical activity and screen time in primary school children: 'Switch Off-Get Active'. J Sci Med Sport.

[152] Hinckson E, Aminian S, Ikeda E, Stewart T, Oliver M, Duncan S, Schofield G (2013). Acceptability of standing workstations in elementary schools: A pilot study. Preventive Medicine.

[153] Hinkley T, Cliff DP, Okely AD. Reducing electronic media use in 2–3 year-old children: feasibility and efficacy of the Family@play pilot randomised controlled trial. BMC Public Health. 2015;15(779). 10.1186/s12889-015-2126-2 PMC4535563 26271928

[154] Kipping R, Payne C, Lawlor D (2008). Randomised controlled trial adapting US school obesity prevention to England. Arch Dis Child.

[155] Kipping, R., Howe, L., Jago, R., Campbell, R., Wells, S., Chittleborough, C., Lawlor, D. (2014) Effect of intervention aimed at increasing physical activity, reducing sedentary behaviour, and increasing fruit and vegetable consumption in children: Active for Life Year 5 (AFLY5) school based cluster randomised controlled trial. BMJ (Online), 348. 10.1136/bmj.g3256 PMC4035503 24865166

[156] Lubans D, Morgan P, Callister R, Collins C (2009). Effects of integrating pedometers, parental materials, and e-mail support within an extracurricular school sport intervention. J Adolesc Health.

[157] Maloney A, Carter Bethea T, Kelsey K, Marks J, Paez S, Rosenberg A (2008). A pilot of a video game (DDR) to promote physical activity and decrease sedentary screen time. Obesity.

[158] Mendoza J, Baranowski T, Jaramillo S, Fesinmeyer M, Haaland W, Thompson D, Nicklas T (2016). Fit 5 kids TV reduction program for latino preschoolers: A cluster randomized controlled trial. Am J Preventive Med.

[159] Moshki M, Noghabi A, Darabi F, Palangi H, Bahri N. The effect of educational programs based on the theory of planned behavior on parental supervision in students' television watching. Med J Islamic Republic Iran. 2016;30(1). PMC5038991 27683647

[160] Murillo-Pardo B, García Bengoechea E, Generelo Lanaspa E, Zaragoza Casterad J, Julián Clemente J (2015). Effects of the 3-year Sigue la Huella intervention on sedentary time in secondary school students. Eur J Public Health.

[161] Nemet D, Barkan S, Epstein Y, Friedland O, Kowen G, Eliakim A (2005). Short- and long-term beneficial effects of a combined dietary-behavioral-physical activity intervention for the treatment of childhood obesity. Pediatrics.

[162] Ni Mhurchu C, Roberts V, Maddison R, Dorey E, Jiang Y, Jull A, Tin Tin S (2009). Effect of electronic time monitors on children's television watching: Pilot trial of a home-based intervention. Preventive Medicine.

[163] Norris E, Dunsmuir S, Duke-Williams O, Stamatakis E, Shelton N (2018). Physically active lessons improve lesson activity and on-task behavior: A cluster-randomized controlled trial of the “Virtual Traveller” Intervention. Health Educ Behav.

[164] Nyberg G, Sundblom E, Norman Å, Bohman B, Hagberg J, Elinder LS (2015). Effectiveness of a universal parental support programme to promote healthy dietary habits and physical activity and to prevent overweight and obesity in 6-year-old children: the Healthy School Start Study, a cluster-randomised controlled trial. PLoS One.

[165] Nyberg G, Norman Å, Sundblom E, Zeebari Z, Elinder LS. Effectiveness of a universal parental support programme to promote health behaviours and prevent overweight and obesity in 6-year-old children in disadvantaged areas, the Healthy School Start Study II, a cluster-randomised controlled trial. Int J Behav Nutr Physical Act. 2016;13(4). 10.1186/s12966-016-0327-4 PMC4721005 26795378

[166] Parrish A, Trost S, Howard S, Batterham M, Cliff D, Salmon J, Okely A (2018). Evaluation of an intervention to reduce adolescent sitting time during the school day: The 'stand Up for Health’ randomised controlled trial. J Sc Med Sport.

[167] Patrick K, Norman G, Zabinski M, Covin J, Calfas K, Sallis J, Cella J (2006). Randomized controlled trial of a primary care and home-based intervention for physical activity and nutrition behaviors: PACE+ for adolescents. Arch Pediatr Adolesc Med.

[168] Pbert L, Druker S, Barton B, Olendzki B, Andersen V, Persuitte G, Geller A (2016). Use of a FITLINE to support families of overweight and obese children in pediatric practices. Childhood Obes.

[169] Robinson T, Borzekowski D (2006). Effects of the SMART classroom curriculum to reduce child and family screen time. J Commun.

[170] Salmon J, Ball K, Hume C, Booth M, Crawford D (2008). Outcomes of a group-randomized trial to prevent excess weight gain, reduce screen behaviours and promote physical activity in 10-year-old children: Switch-Play. Int J Obes.

[171] Sanders W, Parent J, Forehand R (2018). Parenting to reduce child screen time: A feasibility pilot study. J Dev Behav Pediatr.

[172] Sevil J, García-González L, Abós Á, Generelo E, Aibar A (2019). Can High Schools Be an Effective Setting to Promote Healthy Lifestyles? Effects of a Multiple Behavior Change Intervention in Adolescents. J Adolesc Health.

[173] Shapiro J, Bauer S, Hamer R, Kordy H, Ward D, Bulik C (2008). Use of text messaging for monitoring sugar-sweetened beverages, physical activity, and screen time in children: A pilot study. J Nutr Educ Behav.

[174] Silva C, Fassnacht D, Ali K, Gonçalves S, Conceição E, Vaz A (2015). Promoting health behaviour in Portuguese children via Short Message Service: The efficacy of a text-messaging programme. J Health Psychol.

[175] Silva D, Minderico C, Pinto F, Collings P, Cyrino E, Sardinha L (2018). Impact of a classroom standing desk intervention on daily objectively measured sedentary behavior and physical activity in youth. J Sci Med Sport.

[176] Singh AS, Chin A, Paw MJ, Brug J, van Mechelen W (2009). Dutch Obesity Intervention in Teenagers Effectiveness of a School-Based Program on Body Composition and Behavior. Arch Pediatr Adolesc Med.

[177] Smith J, Morgan P, Lonsdale C, Dally K, Plotnikoff R, Lubans D (2017). Mediators of change in screen-time in a school-based intervention for adolescent boys: findings from the ATLAS cluster randomized controlled trial. J Behav Med.

[178] Spruijt-Metz D, Nguyen-Michel S, Goran M, Chou C, Huang T (2008). Reducing sedentary behavior in minority girls via a theory-based, tailored classroom media intervention. Int J Pediatr Obes.

[179] St George, S. (2014) Project SHINE: A Family-Based Intervention for Improving Physical Activity, Sedentary Behavior, and Diet in African American Adolescents. Scholar Commons.

[180] Swartz A, Tokarek N, Lisdahl K, Maeda H, Strath S, Cho C. Do stand-biased desks in the classroom change school-time activity and sedentary behavior? Int J Environ Res Public Health. 2019;16(6). 10.3390/ijerph16060933 PMC6466009 30875890

[181] Taveras E, Gortmaker S, Hohman K, Horan C, Kleinman K, Mitchell K, Gillman M (2011). Randomized controlled trial to improve primary care to prevent and manage childhood obesity the high five for kids study. Arch Pediatr Adolesc Med.

[182] Taylor S, Noonan R, Knowles Z, Owen M, McGrane B, Curry W, Fairclough S. Evaluation of a pilot school-based physical activity clustered randomised controlled trial—active schools: Skelmersdale. Int J Environ Res Public Health. 2018;15(5). 10.3390/ijerph15051011 PMC5982050 29772839

[183] Todd M, Reis-Bergan M, Sidman C, Flohr J, Jameson-Walker K, Spicer-Bartolau T, Wildeman K (2008). Effect of a family-based intervention on electronic media use and body composition among boys aged 8-11 years: A pilot study. J Child Health Care.

[184] Tuominen PPA, Husu P, Raitanen J, Kujala UM, Luoto RM (2017). The effect of a movement-to-music video program on the objectively measured sedentary time and physical activity of preschool-aged children and their mothers: A randomized controlled trial. PLoS One.

[185] Van Kann D, Kremers S, de Vries N, de Vries S, Jansen M (2016). The effect of a school-centered multicomponent intervention on daily physical activity and sedentary behavior in primary school children: The Active Living study. Preventive Medicine.

[186] Van Lippevelde W, Bere E, Verloigne M, Van Stralen M, De Bourdeaudhuij I, Lien N, Maes L. The role of family-related factors in the effects of the UP4FUN school-based family-focused intervention targeting screen time in 10- to 12-year-old children: The ENERGY project. BMC Public Health. 2014;14(1). 10.1186/1471-2458-14-857 PMC4150942 25134740

[187] Verbestel V, De Henauw S, Barba G, Eiben G, Gallois K, Hadjigeorgiou C, De Bourdeaudhuij I (2015). Effectiveness of the IDEFICS intervention on objectively measured physical activity and sedentary time in European children. Obes Rev.

[188] Verloigne M, Ridgers N, Chinapaw M, Altenburg T, Bere E, Berntsen S, Maes L (2014). The UP4FUN Intervention Effect on Breaking up Sedentary Time in 10- to 12-Year-Old Belgian Children: The ENERGY Project. Pediatr Exerc Sci.

[189] Viitasalo A, Eloranta A, Lintu N, Väistö J, Venäläinen T, Kiiskinen S, Lakka T (2016). The effects of a 2-year individualized and family-based lifestyle intervention on physical activity, sedentary behavior and diet in children. Preventive Med.

[190] Vik F, Lien N, Berntsen S, De Bourdeaudhuij I, Grillenberger M, Manios Y, Bere E. Evaluation of the UP4FUN intervention: A cluster randomized trial to reduce and break up sitting time in European 10-12-year-old children. PLoS One. 2015;10(3). 10.1371/journal.pone.0122612 PMC4380348 25826704

[191] Yilmaz G, Caylan N, Karacan C (2015). An intervention to preschool children for reducing screen time: A randomized controlled trial. Child Care Health Dev.

[192] Shrestha N, Grgic J, Wiesner G, Parker A, Podner H, Bennie JA, Biddle SJ, Pedisic Z (2019). Effectiveness of interventions for reducing non-occupational sedentary behaviour in adults and older adults: a systematic review and meta-analysis. Br J Sports Med.

[193] Healy GN, Winkler EA, Owen N, Anuradha S, Dunstan DW (2015). Replacing sitting time with standing or stepping: associations with cardio-metabolic risk biomarkers. Eur Heart J.

[194] Buckley JP, Hedge A, Yates T, Copeland RJ, Loosemore M, Hamer M, Bradley G, Dunstan DW (2015). The sedentary office: a growing case for change towards better health and productivity Expert statement commissioned by Public Health England and the Ative Working Community Interest Company. Br J Sports Med.

[195] Gardner B, Smith L, Lorencatto F, Hamer M, Biddle SJ (2016). How to reduce sitting time? A review of behaviour change strategies used in sedentary behaviour reduction interventions among adults. Health Psychol Rev.

[196] Brunton G, Harden A, Rees R, Kavanagh J, Oliver S, Oakley A (2003). Children and Physical Activity: A systematic review of barriers and facilitators – Executive Summary.

[197] Downing KL, Hnatiuk JA, Hinkley T, Salmon J, Hesketh KD (2018). Interventions to reduce sedentary behaviour in 0-5-year-olds: a systematic review and meta-analysis of randomised controlled trials. Br J Sports Med.

[198] De Meester F, van Lenthe FJ, Spittaels H, Lien N, De Bourdeaudhuij I. Interventions for promoting physical activity among European teenagers: a systematic review. Int J Behav Nutr Physical Act. 2009;6(82). 10.1186/1479-5868-6-82 PMC2795736 19961623

[199] Salmon J, Booth ML, Phongsavan P, Murphy N, Timperio A (2007). Promoting physical activity participation among children and adolescents. Epidemiol Rev.

[200] van Sluijs EM, McMinn AM, Griffin SJ (2007). Effectiveness of interventions to promote physical activity in children and adolescents: systematic review of controlled trials. Br Med J.

[201] Kriemler S, Meyer U, Martin E, van Sluijs EMF, Andersen LB, Martin BW (2011). Effect of school-based interventions on physical activity and fitness in children and adolescents: a review of reviews and systematic update. Br J Sports Med.

[202] Hegarty LM, Mair JL, Kirby K, Murtagh E, Murphy MH (2016). School-based Interventions to reduce sedentary behaviour in children: A systematic review. AIMS Public Health.

[203] Biddle SJ, Petrolini I, Pearson N (2013). Interventions designed to reduce sedentary behaviours in young peple: a review of reviews. Br J Sports Med.

