# Supplementary material for: The effectiveness and complexity of interventions targeting sedentary behaviour across the lifespan: a systematic review and meta-analysis
Source: Int J Behav Nutr Phys Act. 2020 Apr 25;17:53. doi: 10.1186/s12966-020-00957-0 (PMC7183680; doi:10.1186/s12966-020-00957-0)
Supplement: Supplementary file 1 — Additional file 1: Supplement A. Search Strategy [file 12966_2020_957_MOESM1_ESM.docx]

**Supplement A: Search Strategy**

**MEDLINE**

| **1** | Sedentary lifestyle/ |
| --- | --- |
| **2** | (sedentary adj (lifestyle* or life-style* or behavio?r* or time)).ti,ab. |
| **3** | ((sitting or lying) adj2 time).ti,ab. |
| **4** | Screen time.ti,ab. |
| **5** | media time.ti,ab. |
| **6** | ((television or TV) adj (viewing or watching)).ti,ab. |
| **7** | ((computer or internet or driving) adj3 time).ti,ab. |
| **8** | ((computer or internet) adj us*).ti,ab. |
| **9** | ((computer or video) adj game*).ti,ab. |
| **10** | ((screen or screen-based) adj (entertainment or behavio?r* or us*)).ti,ab. |
| **11** | Low energy expenditure*.ti,ab. |
| **12** | sitting.ti,ab. |
| **13** | Or/1-12 |
| **14** | Intervention studies/ |
| **15** | Intervention*.ti,ab. |
| **16** | exp health promotion/ |
| **17** | Health education/ |
| **18** | (health$ adj3 (promot$ or educat$ or lifestyle)).ti,ab. |
| **19** | behavior therapy/ |
| **20** | behavio?r change.ti,ab. |
| **21** | Or/14-20 |
| **22** | Cost benefit analysis/ |
| **23** | Cost adj2 analys*.ti,ab. |
| **24** | Economic evaluation.ti,ab. |
| **25** | Quality adjusted life year/ |
| **26** | Q?aly.ti,ab. |
| **27** | or/22-26 |
| **28** | 21 or 27 |
| **29** | 13 and 28 |
| **30** | exp animals/ not humans.sh. |
| **31** | 29 not 30 |

**PSYCINFO**

| 1 | Sedentary lifestyle/ |
| --- | --- |
| 2 | (sedentary adj (lifestyle* or life-style* or behavio?r* or time)).ti,ab. |
| 3 | ((sitting or lying) adj2 time).ti,ab. |
| 4 | Screen time.ti,ab. |
| 5 | media time.ti,ab. |
| 6 | ((television or TV) adj (viewing or watching)).ti,ab. |
| 7 | ((computer or internet or driving) adj3 time).ti,ab. |
| 8 | ((computer or internet) adj us*).ti,ab. |
| 9 | ((computer or video) adj game*).ti,ab. |
| 10 | ((screen or screen-based) adj (entertainment or behavio?r* or us*)).ti,ab. |
| 11 | Low energy expenditure*.ti,ab. |
| 12 | sitting.ti,ab. |
| 13 | or/1-12 |
| 14 | intervention/ |
| 15 | Intervention*.ti,ab. |
| 16 | exp health promotion/ |
| 17 | Health education/ |
| 18 | (health$ adj3 (promot$ or educat$ or lifestyle)).ti,ab. |
| 19 | behavior therapy/ |
| 20 | behavio?r change.ti,ab. |
| 21 | or/14-20 |
| 22 | "Costs and Cost Analysis"/ |
| 23 | (Cost adj2 analys*).ti,ab. |
| 24 | Economic evaluation.ti,ab. |
| 25 | Q?aly.ti,ab. |
| 26 | or/22-25 |
| 27 | 21 or 26 |
| 28 | 13 and 27 |
| 29 | exp animals/ not humans.sh. |
| 30 | 28 not 29 |

**WEB OF SCIENCE**

| # 22 | #21 AND #12 |
| --- | --- |
|  | *Indexes=SCI-EXPANDED, SSCI, CPCI-SSH Timespan=All years* |
| # 21 | #20 OR #16 |
|  | *Indexes=SCI-EXPANDED, SSCI, CPCI-SSH Timespan=All years* |
| # 20 | #19 OR #18 OR #17 |
|  | *Indexes=SCI-EXPANDED, SSCI, CPCI-SSH Timespan=All years* |
| # 19 | TI=Q*aly |
|  | *Indexes=SCI-EXPANDED, SSCI, CPCI-SSH Timespan=All years* |
| # 18 | TI=Economic evaluation |
|  | *Indexes=SCI-EXPANDED, SSCI, CPCI-SSH Timespan=All years* |
| # 17 | TI=(Cost NEAR/2 analys*) |
|  | *Indexes=SCI-EXPANDED, SSCI, CPCI-SSH Timespan=All years* |
| # 16 | #15 OR #14 OR #13 |
|  | *Indexes=SCI-EXPANDED, SSCI, CPCI-SSH Timespan=All years* |
| # 15 | TI=behavior change |
|  | *Indexes=SCI-EXPANDED, SSCI, CPCI-SSH Timespan=All years* |
| # 14 | TI=(health* NEAR/3 (promot* or educat* or lifestyle)) |
|  | *Indexes=SCI-EXPANDED, SSCI, CPCI-SSH Timespan=All years* |
| # 13 | TI=Intervention* |
|  | *Indexes=SCI-EXPANDED, SSCI, CPCI-SSH Timespan=All years* |
| # 12 | #11 OR #10 OR #9 OR #8 OR #7 OR #6 OR #5 OR #4 OR #3 OR #2 OR #1 |
|  | *Indexes=SCI-EXPANDED, SSCI, CPCI-SSH Timespan=All years* |
| # 11 | TI=sitting |
|  | *Indexes=SCI-EXPANDED, SSCI, CPCI-SSH Timespan=All years* |
| # 10 | TI=Low energy expenditure* |
|  | *Indexes=SCI-EXPANDED, SSCI, CPCI-SSH Timespan=All years* |
| # 9 | TI= ((screen or screen-based) NEAR (entertainment or behavior* or use*)) |
|  | *Indexes=SCI-EXPANDED, SSCI, CPCI-SSH Timespan=All years* |
| # 8 | TI=((computer or video) NEAR game*) |
|  | *Indexes=SCI-EXPANDED, SSCI, CPCI-SSH Timespan=All years* |
| # 7 | TI=((computer or internet) NEAR use*) |
|  | *Indexes=SCI-EXPANDED, SSCI, CPCI-SSH Timespan=All years* |
| # 6 | TI=((computer or internet or driving) NEAR/3 time) |
|  | *Indexes=SCI-EXPANDED, SSCI, CPCI-SSH Timespan=All years* |
| # 5 | TI=((television or TV) NEAR (viewing or watching)) |
|  | *Indexes=SCI-EXPANDED, SSCI, CPCI-SSH Timespan=All years* |
| # 4 | TI=(media time) |
|  | *Indexes=SCI-EXPANDED, SSCI, CPCI-SSH Timespan=All years* |
| # 3 | TI=(Screen time) |
|  | *Indexes=SCI-EXPANDED, SSCI, CPCI-SSH Timespan=All years* |
| # 2 | TI= ((sitting or lying) NEAR/2 time) |
|  | *Indexes=SCI-EXPANDED, SSCI, CPCI-SSH Timespan=All years* |
| # 1 | TI=(sedentary NEAR (lifestyle* or life-style* or behavior* or time)) |
|  | *Indexes=SCI-EXPANDED, SSCI Timespan=All years* |

**EMBASE**

| 1 | Sedentary lifestyle/ |
| --- | --- |
| 2 | (sedentary adj (lifestyle* or life-style* or behavio?r* or time)).ti,ab. |
| 3 | ((sitting or lying) adj2 time).ti,ab. |
| 4 | Screen time.ti,ab. |
| 5 | media time.ti,ab. |
| 6 | ((television or TV) adj (viewing or watching)).ti,ab. |
| 7 | ((computer or internet or driving) adj3 time).ti,ab. |
| 8 | ((computer or internet) adj us*).ti,ab. |
| 9 | ((computer or video) adj game*).ti,ab. |
| 10 | ((screen or screen-based) adj (entertainment or behavio?r* or us*)).ti,ab. |
| 11 | Low energy expenditure*.ti,ab. |
| 12 | sitting.ti,ab. |
| 13 | Or/1-12 |
| 14 | Intervention studies/ |
| 15 | Intervention*.ti,ab. |
| 16 | exp health promotion/ |
| 17 | Health education/ |
| 18 | (health$ adj3 (promot$ or educat$ or lifestyle)).ti,ab. |
| 19 | behavior therapy/ |
| 20 | behavio?r change.ti,ab. |
| 21 | Or/14-20 |
| 22 | Cost benefit analysis/ |
| 23 | Cost adj2 analys*.ti,ab. |
| 24 | Economic evaluation.ti,ab. |
| 25 | Quality adjusted life year/ |
| 26 | Q?aly.ti,ab. |
| 27 | or/22-26 |
| 28 | 21 or 27 |
| 29 | 13 and 28 |
| 30 | exp animals/ not humans.sh. |
| 31 | 29 not 30 |

**PHYSICAL EDUCATION INDEX**

| **#** | **SEARCH TERMS** | **FIELD** |
| --- | --- | --- |
| S1 | (Sedentary lifestyle) | Subject Heading |
| S2 | (sedentary N1 (lifestyle* or life-style* or behavio#r* or or time)) or ((sitting or lying) N2 time) or (Screen time) or (media time) or ((television or TV) N1 (viewing or watching)) or ((computer or internet or driving) N3 time) or ((computer or internet) N1 us*) or ((computer or video) N1 game*) or ((screen or screen-based) N1 (entertainment or behavio#r* or us*)) or (Low energy expenditure*) or (sitting) | Document Title |
| S3 | (sedentary N1 (lifestyle* or life-style* or behavio#r* or or time)) or ((sitting or lying) N2 time) or (Screen time) or (media time) or ((television or TV) N1 (viewing or watching)) or ((computer or internet or driving) N3 time) or ((computer or internet) N1 us*) or ((computer or video) N1 game*) or ((screen or screen-based) N1 (entertainment or behavio#r* or us*)) or (Low energy expenditure*) or (sitting) | Abstract |
| S4 | Intervention studies | Subject Heading |
| S5 | ((Intervention*) OR (health* N3 (promot* OR educat* OR lifestyle)) OR (behavior#r change) OR (Cost N2 anwalys*) OR (Economic evaluation) OR (q#waly)) | Document Title |
| S6 | ((Intervention*) OR (health* N3 (promot* OR educat* OR lifestyle)) OR (behavior#r change) OR (Cost N2 anwalys*) OR (Economic evaluation) OR (q#waly)) | Abstract |
| S7 | Health promotion | Subject heading |
| S8 | Health education | Subject heading |
| S9 | Behavior therapy | Subject heading |
| S10 | Cost benefit analysis | Subject heading |
| S11 | Quality adjusted life year | Subject heading |
| S12 | (S1 or S2 or S3)  su((Sedentary lifestyle)) OR ti((sedentary N1 (lifestyle* or life-style* or behavio#r* or or time)) or ((sitting or lying) N2 time) or (Screen time) or (media time) or ((television or TV) N1 (viewing or watching)) or ((computer or internet or driving) N3 time) or ((computer or internet) N1 us*) or ((computer or video) N1 game*) or ((screen or screen-based) N1 (entertainment or behavio#r* or us*)) or (Low energy expenditure*) or (sitting)) OR ab((sedentary N1 (lifestyle* or life-style* or behavio#r* or or time)) or ((sitting or lying) N2 time) or (Screen time) or (media time) or ((television or TV) N1 (viewing or watching)) or ((computer or internet or driving) N3 time) or ((computer or internet) N1 us*) or ((computer or video) N1 game*) or ((screen or screen-based) N1 (entertainment or behavio#r* or us*)) or (Low energy expenditure*) or (sitting)) |  |
| S13 | (S4 or S5 or S6 or S7 or S8 or S9 or S10 or S11)  su(Intervention studies) OR ti(((Intervention*) OR (health* N3 (promot* OR educat* OR lifestyle)) OR (behavior#r change) OR (Cost N2 anwalys*) OR (Economic evaluation) OR (q#waly))) OR ab(((Intervention*) OR (health* N3 (promot* OR educat* OR lifestyle)) OR (behavior#r change) OR (Cost N2 anwalys*) OR (Economic evaluation) OR (q#waly))) OR su(Health promotion) OR su(Health education) OR su(Behavior therapy) OR su(Cost benefit analysis) OR su(Quality adjusted life year) |  |
| S14 | S12 and S13  (su((Sedentary lifestyle)) OR ti((sedentary N1 (lifestyle* or life-style* or behavio#r* or or time)) or ((sitting or lying) N2 time) or (Screen time) or (media time) or ((television or TV) N1 (viewing or watching)) or ((computer or internet or driving) N3 time) or ((computer or internet) N1 us*) or ((computer or video) N1 game*) or ((screen or screen-based) N1 (entertainment or behavio#r* or us*)) or (Low energy expenditure*) or (sitting)) OR ab((sedentary N1 (lifestyle* or life-style* or behavio#r* or or time)) or ((sitting or lying) N2 time) or (Screen time) or (media time) or ((television or TV) N1 (viewing or watching)) or ((computer or internet or driving) N3 time) or ((computer or internet) N1 us*) or ((computer or video) N1 game*) or ((screen or screen-based) N1 (entertainment or behavio#r* or us*)) or (Low energy expenditure*) or (sitting))) AND (su(Intervention studies) OR ti(((Intervention*) OR (health* N3 (promot* OR educat* OR lifestyle)) OR (behavior#r change) OR (Cost N2 anwalys*) OR (Economic evaluation) OR (q#waly))) OR ab(((Intervention*) OR (health* N3 (promot* OR educat* OR lifestyle)) OR (behavior#r change) OR (Cost N2 anwalys*) OR (Economic evaluation) OR (q#waly))) OR su(Health promotion) OR su(Health education) OR su(Behavior therapy) OR su(Cost benefit analysis) OR su(Quality adjusted life year)) |  |

**PROQUEST**

| **#** | **SEARCH TERMS** | **FIELD** |
| --- | --- | --- |
| S1 | (Sedentary lifestyle) | Subject Heading |
| S2 | (sedentary N1 (lifestyle* or life-style* or behavio#r* or or time)) or ((sitting or lying) N2 time) or (Screen time) or (media time) or ((television or TV) N1 (viewing or watching)) or ((computer or internet or driving) N3 time) or ((computer or internet) N1 us*) or ((computer or video) N1 game*) or ((screen or screen-based) N1 (entertainment or behavio#r* or us*)) or (Low energy expenditure*) or (sitting) | Document Title |
| S3 | (sedentary N1 (lifestyle* or life-style* or behavio#r* or or time)) or ((sitting or lying) N2 time) or (Screen time) or (media time) or ((television or TV) N1 (viewing or watching)) or ((computer or internet or driving) N3 time) or ((computer or internet) N1 us*) or ((computer or video) N1 game*) or ((screen or screen-based) N1 (entertainment or behavio#r* or us*)) or (Low energy expenditure*) or (sitting) | Abstract |
| S4 | Intervention studies | Subject Heading |
| S5 | ((Intervention*) OR (health* N3 (promot* OR educat* OR lifestyle)) OR (behavior#r change) OR (Cost N2 anwalys*) OR (Economic evaluation) OR (q#waly)) | Document Title |
| S6 | ((Intervention*) OR (health* N3 (promot* OR educat* OR lifestyle)) OR (behavior#r change) OR (Cost N2 anwalys*) OR (Economic evaluation) OR (q#waly)) | Abstract |
| S7 | Health promotion | Subject heading |
| S8 | Health education | Subject heading |
| S9 | Behavior therapy | Subject heading |
| S10 | Cost benefit analysis | Subject heading |
| S11 | Quality adjusted life year | Subject heading |
| S12 | (S1 or S2 or S3) | Anywhere except full text |
| S13 | (S4 or S5 or S6 or S7 or S8 or S9 or S10 or S11) | Anywhere except full text |
| S14 | S12 and S13 | Anywhere except full text |
