# Supplementary material for: The effectiveness and complexity of interventions targeting sedentary behaviour across the lifespan: a systematic review and meta-analysis
Source: Int J Behav Nutr Phys Act. 2020 Apr 25;17:53. doi: 10.1186/s12966-020-00957-0 (PMC7183680; doi:10.1186/s12966-020-00957-0)
Supplement: Supplementary file 2 — Additional file 2: Supplement B. Characteristics of Included Studies [file 12966_2020_957_MOESM2_ESM.docx]

**Characteristics of Included Studies of Sedentary Behaviour Interventions in Adults (n= 77)**

| **Study ID** | **Sample Size** | **Age** | **Study Design** | **Intervention Type** | **Setting** | **Sedentary Outcome** | **Outcome Measure** | **Length of Intervention** | **Underlying Theory** | **Control** |
| --- | --- | --- | --- | --- | --- | --- | --- | --- | --- | --- |
| Aadahl 2014 (32) | 166  (I=93,63% W)  (C=73, 49% W) | I = 52.2 ± 13.8  C = 51.8 ± 14.3 | RCT | Behavioural | Community | Sitting time (hours/day) | ActivPAL 3TM | 6 months | Behavioural choice theory | Inactive |
| Adams 2013 (33) | 64  (100% female) | I= 56.7 ± 12.64  C= 61.4 ± 12.1 | Quasi-experimental design | Behavioural | Community | Sitting time (hours/week) | Self-report | 6 weeks | Social Cognitive Theory | Inactive |
| Aguinaga 2017 (34) | 21 | 75.4 ± 6.3 | RCT | Behavioural | Community | Sitting time (hours/day) | ActivPAL 3TM | 16 weeks | Not stated | Inactive |
| Alkhajah 2012 (35) | 32  (29 F 3 M) | I= 33.5 ± 8.7  C= 39.9 ± 7.2 | Quasi-experimental design | Environmental | Work | Sitting time (mins/16-hour day) | ActivPAL3 activity monitor | 3 months | Not stated | Inactive |
| Arrogi 2017a (36) | 300  (I=246, C=54)  78% F | 42 ± 9 | Quasi-experimental design | Behavioural | Work | Sedentary time (mins/day) | SenseWear Pro3  IPAQ | 3 months | Self-determination theory | Inactive |
| Arrogi 2017b (37) | 58  I=31, C=27 | I = 33.6 ± 8.3  C = 39.2 ± 11.5 | RCT | Behavioural | Community | Total sitting time (mins/day) | ActivPAL 3 | 1 week | Social cognitive theory | Inactive |
| Balducci 2019 (38) | 267  I = 133  C = 134 | 61.6 ± 8.5 | RCT | Behavioural | Primary Care | Sedentary time (hours/day) | MyWellness Key accelerometer | 3 years | Not stated | Active |
| Barbieri 2017 (39) | 24  16 F 8 M | 41.3 ± 8.8 | RCT | Environmental | Work | Mean duration of uninterrupted sitting (minutes) | Custom-made interface software recorded, and time stamped every change of table position | 2 months | Not stated | Active |
| Barone Gibbs 2017 (40) | 38  71% F | 68 ± 7 | RCT | Behavioural | Community | Sedentary time (hrs/day) | SenseWear armband | 12 weeks | Not stated | Active |
| Barone Gibbs 2018 (41) | 27  I = 13  C = 14 | I = 52 ± 9  C = 51 ± 13 | RCT | Mixed | Work | Sitting time (hrs/day) | Self-reported work sitting time | 6 months | Behaviour change theory | Inactive |
| Barwais 2013 (42) | 33  (11 F 22 M) | I= 29.0 ± 4.4  C= 26.4 ± 3.0 | RCT | Behavioural | Community | Sedentary time (hrs/day) | 7-day SLIPA log | 4 weeks | Not stated | Inactive |
| Biddle 2015 (43) | 187  (69% female) | 32.8 ± 5.6 | RCT | Behavioural | Primary care | Sedentary time (hrs/day) | ActiGraph GT3X | Educational workshop followed by 6-week follow-up phone call | Social cognitive theory  Behavioural choice theories | Active |
| Carr 2016 (44) | 54  (70% female) | I= 45.0 ± 10.7  C= 45.2 ± 10.9 | RCT | Mixed | Work | % work time sedentary | GENEActiv accelerometer | 16 weeks | Social Cognitive Theory | Active |
| Chang 2013 (45) | 48  (56.3% female) | 66.4 ± 4.09 | Quasi-experimental  design | Behavioural | Primary care | Sitting time (mins/week) | IPAQ - total sitting time | 8 weeks | Empowerment Theory | Inactive |
| Chau 2014 (46) | 42 | 38 ± 11 | Randomised Cross-over Trial | Environmental | Work | Time at work spent sitting | ActivPAL,  Occupational Sitting and Physical Activity Questionnaire, Workforce sitting questionnaire | 4 weeks | Not stated | Inactive |
| Chau 2016 (47) | I = 16  C = 15 | I = 31.0 ± 10.0  C = 35.1 ± 11.5 | Quasi-experimental design | Environmental | Work | Sitting time (mins)  Sedentary time (mins) | ActivPAL  ActiGraph  Self-reported | 19 weeks | Not stated | Inactive |
| Chiang 2019 (48) | 115  I = 38  CG = 38  UC = 39 | I = 63.1 ± 8.5  CG = 63.8 ± 7.3  UC = 64.3 ± 8.1 | RCT | Behavioural | Primary Care | Sedentary behaviour (mins/week) | IPAQ - Short Form | 12 weeks | Motivational interviewing | Active = CG Inactive =  UC |
| Danquah 2017 (49) | I = 173  C = 144 | 46 ± 10  45 ± 11 | RCT | Mixed | Work | Sitting time (mins/ 8hr day) | ActiGraph GT3X+ | 1 month | Social cognitive theory, Diffusion of Innovation, Goal Setting Theory | Inactive |
| De Cocker 2016 (50) | I = 78  G = 84  C = 51 | 40.5 ± 8.6  40.7 ± 9.7  39.3 ± 9.0 | RCT | Mixed | Work | Total sitting time awake (hrs/day) | ActivPAL | 1 month | Theory of planned behaviour  Self-regulation theory  Self-determination theory | Active = G  Inactive = C |
| De Greef 2010 (51) | 41 (I = 20, C = 21)  (13 F 28 M) | 61.3 ± 6.5 | RCT | Behavioural | Primary care | Sedentary behaviour (mins/day) | ActiGraph model 7164 | 12 weeks | Not stated | Active |
| De Greef 2011 (52) | 92 (I = 60, C = 32)  (69% M) | 62 ± 9 | RCT | Behavioural | Primary care | Sedentary behaviour (mins/day) | Accelerometer | 24 weeks | Not stated | Inactive |
| Donath 2015 (53) | I = 15  C = 16 | I = 45 ± 12  C = 40 ± 10 | RCT | Environmental | Work | Sitting time (hours/week) | ActiGraph wGT3X-BT | 12 weeks | Not stated | Active |
| Dutta 2014 (54) | 28 | 40.4 years | Randomised Cross-over Trial | Environmental | Work | Percentage of time sitting (%) | Modular signal recorder 145, MSR Electronics GmbH | 4 weeks | Not stated | Inactive |
| Edwardson 2018 (55) | I = 77  C = 69 | I = 41.7 ± 11  C = 40.8 ± 11.3 | Cluster RCT | Mixed | Work | Occupational sitting time (average mins/workday) | ActivPAL micro | 12 months | SMArT Work theory which is grounded in Social Cognitive Theory, Organisational Development Theory, Habit Theory, Self-regulation Theory and Relapse Prevention Theory | Inactive |
| English 2016 (56) | I = 19  C = 14 | I = 65.4 ± 12.3  C = 67.8 ± 13.8 | RCT | Behavioural | Home | Total sitting time (mins/day) | ActivPAL3 | 7 weeks | Not stated | Inactive |
| Evans 2012 (57) | I = 14  C = 14 | I = 49 ± 8  C = 39 ± 10 | RCT | Mixed | Work | Total time sitting (hours/day) | ActivPAL | 5 days | Not stated | Active |
| Fanning 2016 (58) | I = 103  C = 118 | I = 70.12 ± 4.79  C = 71.16 ± 4.62 | RCT | Behavioural | Home | Sedentary time (mins/day) | ActiGraph GT1M or GT3X | 6 months | Not stated | Inactive |
| Frank 2019 (59) | 524  I = 239  C = 285 | I= 46.2  (44.3, 48.1)  C = 44.7  (43.0, 46.4) | Natural Experiment | Environmental | Community | Average daily sedentary time (minutes) | IPAQ – Short Form | Two years | Not stated | Inactive |
| Gao 2016 (60) | I = 24  C = 21 | I = 47.8 ± 10.8  C = 39.0 ± 8.5 | Controlled Before and After Study | Environmental | Work | Sitting time (%) | Self-report computer use at work | 6 months | Not stated | Inactive |
| Gentile 2009 (61) | 1150 parents | Not reported | Cluster RCT | Mixed | Community, School and Home | Screen time (hrs/week) | Self/parent-reported | 6 months | Social ecological framework | Inactive |
| Gilson 2009 (62) | 179  (141 F 38 M) | 41.3 ± 10.1 | RCT | Behavioural | Work | Sitting time (mins/day) | Self-reported sitting time | 10 weeks | Ecological approach | Inactive |
| Graves 2015 (63) | I = 26  C = 21 | I = 38.8 ± 9.8  C = 38.4 ± 9.3 | RCT | Environmental | Work | Sitting time (mins/8hr-workday) | Self-report Ecological momentary assessment diaries | 8 weeks | Not stated | Inactive |
| Hallman 2018 (64) | I = 79  C = 31 | I = 47.5 ± 8.9  C = 45.9 ± 8.9 | Controlled Before and After Study | Environmental | Work | Total sitting time (% working time) | ActiGraph GT3X+ | 3 months | Not stated | Inactive |
| Haslam 2019 (65) | I = 431  C = 218 | 42.2 ± 10.3 | Quasi-experimental Design | Mixed | Work | Total sitting time (mins/day) | Domain Specific Sitting Time Questionnaire | 12 months | Stage of Change Model | Inactive |
| Healy 2016 (66) | I = 136  C = 95 | I = 44.6 ± 9.1  C = 47.0 ± 9.7 | Cluster RCT | Mixed | Work | Sitting time (mins/16hr-day) | ActivPAL 3 | 12 months | Social cognitive theory  Ecological model of sedentary behaviour | Inactive |
| Jago 2013 (67) | 48  (47 F 1 M) | No age of parents | RCT | Behavioural | Community | Parent screen-viewing (% <2hrs screen time / ≥2hrs screen time) | TV viewing questionnaire | 8 weeks | Self-determination Theory | Inactive |
| Júdice 2015 (68) | 10  (5 F 5 M) | 50.4 ± 11.5 | Randomised Cross-over Trial | Behavioural | Work | Sitting time (hours/day) | ActivPAL | 1 week | Not stated | Inactive |
| Kerr 2016 (69) | 30  I = 15  C = 15 | I = 61.0 ± 6.0  C = 60 ± 6.0 | RCT | Behavioural | Community | Average daily sitting minutes | ActivPAL | 2 weeks | Behaviour change strategies and Social ecological model | Active |
| Klaren 2014 (70) | I = 33  C = 37 | I = 49.4 ± 9.2  C = 50.3 ± 9.1 | RCT | Behavioural | Home (Online) | Daily sedentary time (minutes) | IPAQ | 6 months | Social cognitive theory | Inactive |
| Knowlden 2016 (71) | 57 | 35.8 ± 7.48 | RCT | Behavioural | Home | Screen time (mins/day) | Self-reported screen time | 4 weeks | Social Cognitive Theory | Active |
| Kozey-Keadle 2013 (72) | EX=20  EX-rST=20  rST=18  C=10 | 43.9 ± 9.7  42.4 ± 10.7  44.5 ± 9.5  42.7 ± 10.1 | Quasi-experimental design | Behavioural | Community | Sedentary time (%) | ActivPAL | 12 weeks | Not stated | Active = EX Inactive = C |
| Larouche 2018 (73) | 19 | 39.4 ± 10.74 | Randomised Cross-over Trial | Mixed | Work | Sitting time (mins/day) | ActivPAL micro accelerometer | 4 weeks | Social Cognitive Theory | Active |
| Li 2017 (74) | I = 17  C = 9 | 42 ± 11  41 ± 8 | RCT | Environmental | Work | Sitting (min/8hr workday) | ActivPAL | 4 weeks | Not stated | Inactive |
| Lin 2018 (75) | I = 51  C = 50 | 49.5 | Quasi-experimental design | Mixed | Work | Sitting (hours/day) | OSPAQ | 3 months | McLeroy’s ecological model,  Bandura’s self-efficacy theory and Bandura’s social cognitive theory of self-regulation | Inactive |
| Lynch 2019 (76) | I = 43  C = 40 | I = 61.3 ± 5.9  C = 61.9 ± 7.0 | RCT | Behavioural | Community | Sitting time (mins/day) | ActiGraph & ActivPAL | 12 weeks | Not stated | Inactive |
| MacEwan 2017 (77) | I = 15  C = 10 | 43.2 ± 9.7  48.9 ± 11.4 | RCT | Environmental | Work | Workday sitting (mins/day) | ActivPAL | 12 weeks | Not stated | Inactive |
| Maher 2017 (78) | I = 24  C = 17 | 76.9 ± 9.2  (total sample) | Cluster RCT | Behavioural | Community | Sedentary behaviour (mins/workday) | Self-reported measure of weekday and weekend average sedentary behaviour | 2 weeks | HAPA - Health action process approach (social cognitive theoretical model) + habit dual-process framework) | Inactive |
| Mitchell 2019 (79) | I = 85  C = 86 | I = 51.7 ± 12.8  C = 49.5 ± 12.2 | RCT | Behavioural | Community | Sedentary time (mins/day) | GENEActiv | 12 weeks | Social Cognitive Theory, goal setting and self-determination theory | Active |
| Neuhaus 2014 (80) | MC = 16  WO = 14  C = 14 | 37.3 ± 10.7  43.0 ± 10.2  48 ± 11.6 | Cluster RCT | Mixed | Work | Workplace sitting time (hours/8hr workday) | ActivPAL3 | 12 weeks | Social Cognitive Theory | Active = W Inactive = UP |
| O’Dolan 2018 (81) | I = 8  C = 9 | I = 36 (29-42)  C = 42 (22-56) | Cluster RCT | Mixed | Work | Total sitting all day (%) | ActivPAL | 10 weeks | Social Cognitive Theory | Active |
| Orme 2018 (82) | I1 = 12  I2 = 10  C = 11 | 71 ± 20 | RCT | Behavioural | Primary Care | Daily sitting time (mins/day) | Self-report | 2 weeks | Not stated | Active = E Inactive = C |
| Overgaard 2018 (83) | I = 23  C = 20 | I = 45.0 ± 11.5  C = 46.1 ± 10.3 | RCT | Behavioural | Community | Sedentary time  (hours/day) | ActivPAL | 4 weeks | Not stated | Active |
| Parry 2013 (84) | 62  (50 F 12 M) | 43.5 ± 6.4 | Cluster RCT | Mixed | Work | Total sedentary time (% wear time) | ActiGraph GT3X | 12 weeks | Not stated | Active = B+C |
| Pedersen 2013 (85) | 34  (26 F 8 M) | I = 41.5 ± 12.39  C = 43.88 ± 9.65 | RCT | Behavioural | Work | METs (per day sitting) | Self-reported workplace daily energy expenditure | 13 weeks | Not stated | Inactive |
| Prince 2018 (86) | I = 19  C = 21 | 62 ± 10 | RCT | Behavioural | Primary Care | Sedentary time (mins/day) | ActivPAL3 | 8 weeks | Not stated | Inactive |
| Pronk 2012 (87) | 34 (I= 96% F, C=80% F) | I = 38.4 ± 11.4  C = 44.2 ± 11.9 | Interrupted Time Series | Environmental | Work | Time spent sitting at work | Experience sampling methodology | 4 weeks | Not stated | Inactive |
| Puig Ribera 2015 (88) | I = 129  C = 135 | 41 ± 9  43 ± 11 | Quasi-experimental design | Mixed | Work | Sitting time (mins/8hr workday) | International Physical Activity Questionnaire short form | 19 weeks | Not stated | Inactive |
| Raynor 2013a (89) | 24 | I = 53.3 ± 8.0  C = 51.7 ± 10.0 | RCT | Behavioural | Home | TV viewing time (hrs/day) | TV allowances | 8 weeks | Not stated | Active |
| Raynor 2013b (89) | 28 | I = 54.9 ± 7.4  C = 53.3 ± 9.1 | RCT | Behavioural | Home | TV viewing time (hrs/day) | TV allowances | 8 weeks | Not stated | Active |
| Schuna 2014 (90) | I = 21  C = 20 | 40.0 ± 9.5  40.3 ± 10.9 | RCT | Mixed | Work | Sedentary time (mins/hour) | ActiGraph GT3X+ | 3 months | Not stated | Inactive |
| Spring 2012 (91) | 204 | 32.8 ± 11 | RCT | Behavioural | Community | Sedentary leisure (mins/day) | Self-reported | 3 weeks | Behavioural choice theory | Active = PA |
| Spring 2018 (92) | I1 = 84  I2 = 84  C = 44 | I1 = 40.7 ± 11.9  I2 = 40.9 ± 12.5  C = 40.8 ± 10.9 | RCT | Behavioural | Community | Sedentary Leisure Screen Time | Self-reported | 9 months | Not stated | Active |
| Steeves 2012 (93) | 58  (46 F 12 M) | 52.0 ± 8.6 | RCT | Behavioural | Community | TV time (hrs/day) | Self-report activity log | 6 months | Not stated | Active |
| Stephens 2014 (94) | I = 21  C = 22 | 42.2 ± 10.6  42.9 ± 10.3 | Cluster RCT | Mixed | Work | Total sitting time (mins) | ActivPAL 3 | 4 weeks | Not stated | Inactive |
| Sui 2018 (95) | I = 28  C = 24 | 23.52 ± 4.6 | RCT | Behavioural | University | Self-reported sitting time | SIT-Q 7d Questionnaire | 8 weeks | Health Action Process Approach | Inactive |
| Schwartz 2019 (96) | I = 12  C = 6 | I = 35.7 ± 9.6  C = 37.5 ± 12.5 | Randomised Cross-over Trial | Environmental | Work | Occupational sitting time (hours/day) | IPAQ | 23 weeks | Not stated | Inactive |
| Taylor 2016 (97) | BB = 76  P = 61  C = 48 | 43 | Cluster RCT | Behavioural | Work | Total sedentary time (mins/week) | International physical activity questionnaire long version and the Neighbourhood quality of life study | 6 months | Not stated | Inactive |
| Ter Hoeve 2018 (98) | CR+F = 161  CR+T = 165  CR = 163 | CR+F = 58.8 ± 9  CR+T = 58.2 ± 9  CR = 59.1 ± 8 | RCT | Behavioural | Primary Care | Sedentary behaviour (% of wear time) | ActiGraph | 12 months | Not stated | Inactive = CR |
| Thomsen 2016 (99) | I = 10  C = 10 | 64.5 ± 8.5  54.0 ± 14.0 | RCT | Behavioural | Primary care | Daily sitting time (hours/day) | ActivPAL 3TM | 16 weeks | Behavioural choice theory | Inactive |
| Thomsen 2017 (100) | I = 75  C = 75 | 60 years | RCT | Behavioural | Primary care | Daily sitting time (hours/day) | ActivPAL 3TM | 16 weeks | Behavioural choice theory | Inactive |
| Tobin 2016 (101) | I = 18  C = 19 | 34.8 ± 10.5  34.3 ± 8.9 | RCT | Environmental | Work | Sitting time (mins/8hr workday) | ActivPAL | 3 weeks | Not stated | Inactive |
| Tuominen 2017 (102) | Mother I = 101  C = 102 | 37.0 ± 4.7  37.9 ± 5.0 | RCT | Behavioural | Home | (%) Proportion of sedentary behaviour | Hookie AM20 | 7 weeks | Not stated | Inactive |
| Urda 2016 (103) | I = 22  C = 22 | 48 ± 10  (total sample) | RCT | Behavioural | Work | Workplace sitting time (hrs/workday) | ActivPAL 3 | 1 week | Not stated | Inactive |
| Verweij 2012 (104) | I = 274  C = 249 | 46 ± 8  48 ± 9 | RCT | Mixed | Primary Care | Total sedentary behaviour (mins/day) | Self-reported and IPAQ | 6 months | Not stated | Inactive |
| Whaley 2010 (105) | 821  I=412 C= 409 | No age of parents | Controlled Before and After Study | Behavioural | Home | TV watching (hrs/day) | Parent-reported TV time | 12 months | The Transtheoretical Model | Inactive |
| Wyke 2019 (106) | I = 560  C = 553 | I = 45.9 ± 9.0  C = 45.6 ± 8.7 | RCT | Behavioural | Community | Sedentary time (mins/day) | ActivPAL | 12 weeks | Not stated | Inactive |
| Zhu 2018 (107) | I = 24  C = 12 | I = 41.3 ± 11.6  C = 34.8 ± 9.9 | Controlled Before and After Study | Mixed | Work | Sitting time (mins/8hr workday) | ActivPAL 3c | 4 months | Not stated | Active |

Abbreviations: C = control; EX = exercise; F = female; hr = hour; hrs = hours; I = intervention; IPAQ = International Physical Activity Questionnaire; M = male; METS = metabolic equivalents; mins = minutes; OSPAQ = occupational sitting and physical activity questionnaire; RCT = randomised controlled trial; SIT-Q-7d = last 7-d sedentary behavior questionnaire; UC = usual care

**Characteristics of Included Studies of Sedentary Behaviour Interventions in Children (n=84)**

| **Study ID** | **Sample Size** | **Age** | **Study Design** | **Intervention Type** | **Setting** | **Sedentary Outcome** | **Outcome Measure** | **Length of Intervention** | **Underlying Theory** | **Control** |
| --- | --- | --- | --- | --- | --- | --- | --- | --- | --- | --- |
| Aittasalo 2019 (108) | I = 690  C = 860 | I = 13.0 ± 0.5  C = 13.9 ± 0.5 | Cluster RCT | Behavioural | School | Sedentary behaviour during school hours (mins/day) | Self-reported Activity Diary | 3 weeks | Health Action Process Model - Behaviour change theory | Inactive |
| Andrade 2014 (109) and 2015 (110) | 1440  I = 700  C = 740 | 12.9 ± 0.8 | Cluster RCT | Mixed | School | Sedentary time (mins/day) | GT-256 and GT1M ActiGraph /  Self-reported screen time | 28 months | Social cognitive theory, information-motivation behavioural skills model, control theory, transtheoretical model and theory of planned behaviour | Inactive |
| Ayala 2017 (111) | I = 20  C = 21 | 11.5 ± 0.34  11.7 ± 0.29 | Cluster Controlled Before and After Study | Environmental | School | Sedentary time (mins/day) | ActivPAL | 8 months | Not stated | Inactive |
| Babic 2016 (112) | 322  I =167  C = 155 | 14.4 ± 0.6 | Cluster RCT | Mixed | School/Home | Recreational screen time (mins/day) | Adolescent Sedentary Activity Questionnaire | 6 months | Self-determination theory | Inactive |
| Bergh 2014 (113) | I = 510  C = 908 | I = 11.2 ± 0.26  C = 11.2 ± 0.27 | RCT | Mixed | School | Screen time (hrs/weekend/  weekday) | Self-reported weekday/weekend TV viewing and computer game use | 20 months | Social ecological approach | Inactive |
| Bickham 2018 (114) | I = 157  C = 379 | I = 12.4  C = 12.8 | Quasi-experimental design | Behavioural | School | Media use after school (mins/day) | Self-reported electronic media use | 7.5 weeks | Social Cognitive Theory | Inactive |
| Birken 2012 (115) | I = 81  C = 79 | I = 3.12 (0.19)  C = 3.08 (0.12) | RCT | Mixed | Primary care | Screen time (mins/weekend/ weekday) | Parent reported screen time | 12 months | Embedded concepts of goal setting, positive reinforcement, monitoring and cognitive restructuring (Bandura, 1986) | Inactive |
| Bjelland 2011 (116) | 1465 | I=11.2 ± 0.26  C=11.2 ± 0.27 | RCT | Mixed | School | Watching TV/DVD (hrs/day) | Self-reported screen time | 8 months | Not stated | Inactive |
| Brittin 2017 (117) | I= 21  C= 20 | I = 15% 8yr 85% 9yr  C = 9% 8yr 91% 9yr | Quasi-experimental design | Environmental | School | Sedentary time (mins/day) | ActiGraph GT3X+  ActiGraph GT1M | New school move (14 months between pre and post assessment) | Not stated | Inactive |
| Byun 2018 (118) | I = 48  C = 45 | I = 4.6 ± 0.7  C = =4.8 ± 0.7 | Quasi-experimental design | Behavioural | School | Sedentary time (mins/hour per 8hr day) | ActiGraph GT3X | 1 week | Social Ecological Model | Inactive |
| Carson 2013 (119) | 293  SB = 74  PA = 75  SB+PA = 80  C = 64 | 8.0 ± 1.3 | Cluster RCT | Behavioural | School | Total weekday sedentary time (mins/day) | ActiGraph GT3X | 18 months | Social cognitive theory, behavioural choice theory and ecological systems theory | Active = PA Inactive = C |
| Cespedes 2014 (120) | 445  48% F | 4.9 ± 1.2 | Cluster RCT | Behavioural | Primary care | TV/video viewing (hrs/day) | Parent reported TV/ video viewing (National Longitudinal Study of Youth) | 1 year | Chronic Care Model | Inactive |
| Chesham 2018 (121) | I = 259  C = 132 | I = 8.1 ± 2.0  C = 8.8 ± 1.8 | Quasi-experimental design | Behavioural | School | Sedentary time (mins/day) | ActiGraph (wGT3X-BT, wGT3X+, GT3X+, GT3X and GT1M). | 8 months | Not stated | Inactive |
| Chin 2008 (122) | 854 | FI=12.6±0.4  FC=12.7±0.5  MI=12.7±0.5  MC=12.8±0.5 | Cluster RCT | Mixed | School | Screen viewing behaviour (mins/day) | DOit questionnaire (parent-reported) | 8 months | Dual-process theory, ANGELO model, theory of planned behaviour and habit theory | Inactive |
| Clemes 2015a (123) | I = 16  C = 14 | 10 ± 0.3 | Controlled Before and After Study | Environmental | School | Sitting (mins/day) | ActivPAL | 9 weeks | Not stated | Inactive |
| Clemes 2015b (123) | I = 24  C = 20 | 11.6 ± 0.5 | Controlled Before and After Study | Environmental | School | Sitting (mins/day) | ActivPAL | 10 weeks | Not stated | Inactive |
| D’Haese 2015 (124) | 126 | 9.0 ± 2.1 | Natural Experiment | Environmental | Community | Sedentary time (mins/day) | ActiGraph GT1M, GT3X or GT3X+ accelerometer | 2 months | Not stated | Inactive |
| De Bourdeaudhuji 2015 (125) | 16228  I = 7,747  C = 8,482 | 2 - 9.9 years | Cluster RCT | Mixed | Community | Total screen time (hrs/day) | Parental questionnaire - media consumption | 2 years | Socio economic theory | Inactive |
| De Craemer 2016 (126) | 859 | I = 4.4 ± 0.6  C = 4.3 ± 0.6 | Cluster RCT | Mixed | School | Sedentary behaviour weekday/end (%/day) | ActiGraph GT1M, GT3X or GT3X+ accelerometer | 24 weeks | Socio-ecological model | Inactive |
| De Lepeleere 2017 (127) | I = 104  C = 103 | 9.2 ± 1.5  9.6 ± 1.6 | Quasi experimental design | Behavioural | School | Screen time (mins/day) | Flemish physical activity questionnaire | 4 weeks | Self-determination theory  Social cognitive theory | Inactive |
| Dennison 2004 (128) | I=106  C-116 | I = 3.9 ± 0.07  C = 4.0 ± 0.10 | RCT | Behavioural | School | Watching TV and video/ Playing video or computer games (hrs/week) | Questionnaire (mean daily TV) parent-reported | 6 months | Not stated | Inactive |
| Downing 2018 (129) | I = 30  C = 27 | I = 3.2 ± 0.8  C = 2.9 ± 0.7 | RCT | Behavioural | Home | Sitting time (mins/day) | ActivPAL | 6 weeks | Social Cognitive Theory | Inactive |
| Ee 2018 (130) | 47 | Aged 10-11 years | Randomised Cross-over Trial | Environmental | School | Sitting time (mins/school-day) | ActiGraph GT9X Link | 2 weeks | Not stated | Inactive |
| Ellis 2019 (131) | I = 55  C = 60 | I = 4.1 ± 0.7  C = 4.2 ± 0.6 | Cluster RCT | Mixed | School | Sitting (%/day) | ActivPAL | 12 weeks | Social Cognitive Theory | Inactive |
| Epstein 2000 (132) | 90 families | 10.5 ± 1.2 | RCT | Behavioural | Primary care | Targeted sedentary time (%) | Minnesota leisure time activity survey | 6 months | Not stated | Active |
| Epstein 2004 (133) | I=32  (21 F 11 M)  C=30  (18F 12M) | I= 9.8 ± 1.4  C= 9.9 ± 1.2 | RCT | Mixed | Primary care | Targeted sedentary time (%) | Self-report index cards | 12 months | Behavioural economic theory | Active |
| Epstein 2008 (134) | I = 36 (17 F 19 M)  C = 34 (16 F 18 M) | I = 5.8 ± 1.2  C = 6.1 ± 1.3 | RCT | Mixed | Primary care | TV and computer time (hrs/week) | TV allowance | 24 months | Not stated | Inactive |
| Escobar-Chaves 2010 (135) | 202 | 8.2 ± 0.8 | RCT | Mixed | Home | Media use - total exposure (hrs/day) | Self-report (Self-interview system) | 6 months | Social cognitive theory | Inactive |
| Faith 2001 (136) | 10  (3 F 7 M) | I = 10.2 ± 1.5  C = 10.0 ± 1.6 | RCT | Mixed | Home | TV viewing (hours/week) Micro-computer of the TV cycle | TV viewing recorded by the TV cycle | 12 weeks | Behavioural theory | Active |
| Farley 2007 (137) | 710  I=506  C=204 | Aged 12-19 | Quasi experimental design | Environmental | School | Watching TV, movies, DVDs, Playing video games, compute, doing homework and reading (%) | Self-reported activity | 2 years | Not stated | Inactive |
| Fassnacht 2014 (138) | 49  (26 F 23 M) | 9.6 ± 0.4 | Cluster RCT | Behavioural | School | Screen time (mins/day) | Family eating and activity habits questionnaire | 8 weeks | Social cognitive theory | Inactive |
| Fitzgibbon 2005 (139) | 409  (205 F 204 M) | I = 48.6 ± 7.6  C = 50.8 ± 6.4  (age in months) | RCT | Mixed | School | TV viewing (hrs/day) | Parental reported | 14 weeks | Social learning theory, self-determination theory, transtheoretical model | Inactive |
| Fitzgibbon 2011 (140) | 618  I=325  C=293 | I = 50.7 ± 6.8  C = 51.9 ± 6.3  (age in months) | RCT | Mixed | School | TV viewing and screen time (hrs/day) | Parental reported | 14 weeks | Social learning theory and self-determination theory | Inactive |
| Ford 2002 (141) | 28  (15 F 13 M) | I=9.5 ± 1.4  C=9.6 ± 1.7 | RCT | Mixed | Primary care | Children’s weekly TV, videotape, and video game use (hrs/week) | Self-reported TV viewing | 4 weeks | Social cognitive theory | Inactive |
| Foster 2008 (142) | 1349  (725 F 624 M) | I = 11.13±1.0  C = 11.2± 1.0 | Cluster RCT | Mixed | School | Inactivity (hrs/week) Youth/Adolescent Activity Questionnaire | Self-report - Youth adolescent activity questionnaire | 2 years | Not stated | Inactive |
| French 2016 (143) | I = 25  C = 15 | 9.0 ± 2.2 | RCT | Environmental | Home | Television viewing (hrs/day) | Parental-reported | 6 month | Not stated | Inactive |
| Gentile 2009 (61) | 1288  I = 685  C = 674 | 9.6 ± 0.9 | Cluster RCT | Mixed | Community, School and Home | Screen time (hrs/week) | Self/parent-reported | 6 months | Social ecological framework | Inactive |
| Goldfield 2006 (144) | 30  (17 F 13 M) | I = 10.0± 0.9  C = 10.7 ± 1.4 | RCT | Mixed | Home | Targeted sedentary behaviour: Television viewing (mins/day) | Previous Day Physical Activity Recall (PD-PAR) | 8 weeks | Not stated | Active |
| Gortmaker 1999a (145) | 479  (282 F 197 M) | I=9.2  C=9.1 | Quasi-experimental design | Mixed | School | TV viewing (hrs/day) | 24-hr recall / Food and Activity Survey | 2 years | Social cognitive theory | Inactive |
| Gortmaker 1999b (146) | 1295  (627 F 668 M) | 11.7 ± 0.7 | RCT | Mixed | School | TV viewing (hrs/day) | Questionnaire Television and Video Measure | 21 months | Behaviour choice and social cognitive theory | Inactive |
| Haines 2013 (147) | 121  I = 62, C = 59 | 4.1 ± 1.1 | RCT | Mixed | Home | TV (hrs/day) | Self/parent-reported TV time | 6 months | Not stated | Inactive |
| Harrison 2006 (148) | 312  (135 F 177 M) | I = 10.2± 1.2  C = 10.3± 0.8 | Cluster Controlled Before and After Study | Behavioural | School | Screen time (30 min blocks/day) | Previous Day Physical Activity Recall (PDPAR) | 16 weeks | Social cognitive theory | Inactive |
| Hinckson 2013 (149) | 30  (16 F 14 M) | 10 ± 1 | Cluster Controlled Before and After Study | Environmental | School | Sitting time (hrs/day) | ActivPAL | 4 weeks | Not stated | Inactive |
| Hinkley 2015 (150) | I = 12  C = 10 | 2.94 ± 0.61  2.85 ± 0.63 | RCT | Behavioural | Community | Total electronic media use (mins/day) | Time-use diary | 5 weeks | Social cognitive theory  Family systems theories | Inactive |
| Kipping 2008 (151) | 679 | I = 9.43 ± 0.5  C = 9.40 ± 0.49 | Cluster RCT | Behavioural | School | Screen viewing (mins/day) | Self-report Questionnaire | 5 months | Social cognitive theory and behavioural choice of theories of individual change | Inactive |
| Kipping 2014 (152) | I = 1064  C = 1157 | 9.5 ± 0.3  9.5 ± 0.3 | Cluster RCT | Behavioural | School | Sedentary behaviour (mins/day) | ActiGraph GT3X+ | 6-7 months | Social cognitive theory | Inactive |
| Knowlden 2016 (71) | 57 (F) | 5.18±0.79 | RCT | Mixed | Home | Child’s screen time (mins/day) | Self-reported | 4 weeks | Social Cognitive Theory | Active |
| Lubans 2009 (153) | 124 | 14.1 ± 0.8 | RCT | Behavioural | School | Screen time (hrs/day) TV/ Computer/ Games | Adolescent sedentary behaviour questionnaire | 6 months | Social Cognitive Theory | Active |
| Maloney 2008 (154) | 60 | I = 7.5 ± 0.5  C = 7.6 ± 0.5 | RCT | Behavioural | Home | Sedentary PA (mins/day) | ActiGraph | 10 weeks | Not stated | Inactive |
| Mendoza 2016 (155) | I = 90  C = 70  160 (84M 76 F) | I = 4.5 ± 0.5  C = 4.4 ± 0.6 | Cluster RCT | Behavioural | School | TV viewing (mins/day) | Validated 7 day TV diary | 8 weeks | Social cognitive theory | Inactive |
| Moshki 2016 (156) | I = 60  C = 60 | Children in the first and fifth grade of primary school | Quasi-experimental design | Behavioural | Home | Watching TV (hours/day) | TV watching indexes (parent reported) | 1 month | Theory of planned behaviour | Inactive |
| Murillo-Pardo 2014 (157) | I = 368  C = 314 | Students aged 12-15 | Quasi-experimental design | Mixed | School | Daily sedentary time (mins/day) | ActiGraph model 7164 | 3 years | Self-determination theory | Inactive |
| Nemet 2005 (158) | 46  (20 F 26 M) | I = 11.3 ± 2.8  C = 10.9 ± 1.9 | RCT | Behavioural | Primary care | Screen time (hrs/day) Self-report | Self-report questionnaire | 3 months | Not stated | Inactive |
| Ni Mhurchu 2009 (159) | 29  (11 F 18 M) | 10.4 ± 0.9 | RCT | Mixed | Home | Total screen time (mins/week) Self-report | Self-report questionnaire | 6 weeks | Not stated | Active |
| Norris 2018 (160) | I = 133  C = 131 | I = 8.6 ± 0.49  C = 8.6 ± 0.49 | Cluster RCT | Behavioural | School | Sedentary behaviour (mins/school-day) | ActiGraph GT1M | 6 weeks | Behaviour change theory (COM-B model) | Inactive |
| Nyberg 2015 (161) | I = 129  C = 112 | 6.2 ± 0.3  6.2 ± 0.3 | Cluster RCT | Behavioural | School | Sedentary time (% time/week) | ActiGraph GT3XP | 6 months | Social cognitive theory | Inactive |
| Nyberg 2016 (162) | 378  (191 F 187 M) | 6.3 ± 0.3 | Cluster RCT | Behavioural | School | Sedentary time (mins/week) | ActiGraph GT3XP | 6 months | Social Cognitive Theory | Inactive |
| Paarish 2018 (163) | I = 43  C = 45 | I =14.74±0.75  C= 14.68±0.66 | RCT | Mixed | School | Proportion of day sitting (%) | ActivPAL | 5 months | Social Cognitive Theory | Inactive |
| Patrick 2006 (164) | 819  (438 G 381 B) | 12.7 ± 1.3 | RCT | Behavioural | Primary care | Sedentary behaviour (hrs/day) | Self-reported screen time | 12 months | Behavioural determinant model, Social cognitive theory, Transtheoretical model of behaviour change | Inactive |
| Pbert 2016 (165) | 37 | 9.6 ± 1.5 | Controlled Before and After Study | Mixed | Primary care | Hours watching TV (hrs/day) | Self-reported questionnaire about PA behaviours | 6 weeks | Social Cognitive Theory | Inactive |
| Robinson 2006 (166) | 181 | I = 8.9 ± 0.6  C = 8.9 ± 0.7 | RCT | Mixed | School/Home | TV viewing (hrs/day) | Self-report questionnaire | 6 months | Social Cognitive Theory | Inactive |
| Salmon 2008 (167) | 311  (156 F 150 M) | M = 10yrs 8±5  F = 10yrs 8±4 | RCT | Mixed | School | Screen behaviours (mins/week) | Self-reported screen behaviours | 6 months | Social cognitive theory and behavioural choice theory | Inactive |
| Sanders 2018 (168) | I = 20  C = 19 | I = 9.21  C = 8.25 | RCT | Behavioural | Community | Daily hours of screen time | Parent reported screen time | 2 hours | Not stated | Inactive |
| Sevil 2019 (169) | I = 105  C = 105 | I =13.05±0.59  C =13.07±0.63 | Quasi-experimental design | Mixed | School | Sedentary time (mons/day) | ActiGraph GT3X | Academic year | Social Ecological Model, Self-Determination Theory and Theory of Planned Behaviour | Inactive |
| Shapiro 2008 (170) | 58 | 8.7 ± 2.3 | RCT | Behavioural | Primary care | Screen time (mins/day) | Self-reported screen time | 8 weeks | Not stated | Active |
| Silva 2015 (171) | 136 | I = 9.94±0.46  C = 9.87±0.42 | RCT | Behavioural | School | Screen time (hrs/day) | Questionnaire - Screen time | 8 weeks | Social cognitive theory and behavioural models | Active |
| Silva 2018 (172) | I = 22  C = 27 | 11.8 ± 0.4  11.6 ± 0.5 | Cluster Controlled Before and After Study | Environmental | School | Sitting (mins/15hr weekday, mins/14hr weekend) | ActivPAL | 16 weeks | Not stated | Inactive |
| Singh 2009 (173) | 1108 | I (M)= 12.8±0.5  I(F)=12.6±0.5  C(M)=12.9±0.5  C(F)=12.7± 0.5 | RCT | Mixed | School | Screen viewing behaviour (mins/day) | Self-report Questionnaire | 8 months | Not stated | Inactive |
| Smith 2017 (174) | I = 180  C = 181 | 12.7 ± 0.5 | Cluster RCT | Mixed | School | Recreational screen time (mins/day) | ASAQ | 20 week / 8 month | Self-determination theory and social cognitive theory | Inactive |
| Spruijt-Metz 2008 (175) | 459 | 12.47 ± 0.63 | Cluster RCT | Behavioural | School | TV/Video Games/ Internet (reported in ½ hr blocks) | Questionnaire - PDPAR | 5-7 days | Self-determination theory and theory of meaning behaviour | Inactive |
| St George 2014 (176) | I = 49  C = 40 | 12.49 ± 1.56  12.58 ± 1.26 | RCT | Behavioural | Community | Sedentary behaviour (hrs/week) | Self-report - sedentary behaviour scale | 6 weeks | Family systems theory, social cognitive theory and self-determination theory | Inactive |
| Swartz 2019 (177) | I = 58  C = 39 | 10.2 ± 1.4 | Randomised Cross-over trial | Environmental | School | % of time sedentary in the classroom | ActiGraph GT3X and ActiGraph GT3X-BT | 9 weeks | Not stated | Inactive |
| Taveras 2011 (178) | 445  (215 F 230 M) | 4.9 ± 1.2 | Cluster RCT | Mixed | Primary care | Total TV + video viewing (hrs/day) | Self-report Questionnaire | 24 months | Chronic care model | Inactive |
| Taylor 2018 (179) | I = 114  C = 118 | 9-10 years | Cluster RCT | Mixed | School | Whole day sedentary time (mins/day) | ActiGraph | 8 weeks | Socio-ecological model, youth physical activity promotion model, Theory of expanded, extended, and enhanced opportunities | Inactive |
| Todd 2008 (180) | 22 | I = 10.0 ± 0.8  C = 9.7 ± 1.2 | RCT | Mixed | Home | Electronic media use (mins/day) | Self-reported logbooks | 20 weeks | Not stated | Active |
| Tuominen 2017 (181) | I = 101  C = 102 | 6.5 ± 0.5  6.5 ± 0.5 | RCT | Behavioural | Home | Proportion of sedentary behaviour | Hookie AM20 | 7 weeks | Not stated | Inactive |
| Van Kann 2016 (182) | I = 310  C = 219 | 10.13 ± .68  10.13 ± .70 | Quasi-experimental design | Mixed | School | Sedentary behaviour (%) | ActiGraph GT3X | 12 months | Not stated | Inactive |
| Van Lippevelde 2014 (183) | I = 1569  C = 1578 | 11.3 ± 0.8  11.2 ± 0.8 | RCT | Mixed | School/Home | Children’s screen time (hrs/day) | Self-report Questionnaire | 6 week | Socio-ecological approach | Inactive |
| Verbestel 2015 (184) | 7413  3663 F, 3750 M | IM= 6.22 ± 1.81 IF= 6.26 ± 1.79 CM= 6.12 ±1.73  CF= 6.25 ± 1.7 | Cluster Controlled Before and After Study | Mixed | Community | Proportion of time spent sedentary (% of time) | ActiGraph GT1M and ActiTrainer TM | 2 year | Socio-ecological approach | Inactive |
| Verloigne 2015 (185) | I = 155  C = 199 | 10.9 ± 0.7 | RCT | Behavioural | Home/School | Total sedentary time (mins/day) | ActiGraph | 6 weeks | Theory of planned behaviour | Inactive |
| Viitasalo 2016 (186) | I = 306  C = 200 | 7.6 ± 0.4  7.6 ± 0.4 | Controlled Before and After Study | Behavioural | Community | Screen-based sedentary behaviour (mins/day) | PANIC Physical Activity Questionnaire | 2 year | Not stated | Active |
| Vik 2015 (187) | I = 1713  C = 1681 | 11.2 (overall mean age) | Cluster RCT | Mixed | School/Home | Total sedentary time (hrs/day) | ActiGraph GT1M, GT3X or ActiTrainer | 6 weeks | Socio ecological framework | Inactive |
| Whaley 2010 (105) | 821  I=412 C= 409 | I=23 ± 9.2  C=22 ± 9.0  (age in mths) | Controlled Before and After Study | Behavioural | Home | TV watching (hrs/day) | Parent-reported TV time | 12 months | The Transtheoretical Model | Inactive |
| Yilmaz 2014 (188) | I = 187 (124 F)  C = 176 (114 F) | I = 3.52 ± 1.28  C = 3.49 ± 1.22 | RCT | Behavioural | Primary care | Child media time (mins/week) | Parent reported | 8 weeks | Social cognitive theory | Inactive |

Abbreviations: ASAQ = Adolescent Sedentary Activity Questionnaire; C = control; F = female; hr = hour; hrs = hours; I = intervention; M = male; mins = minute; mins = minutes; mts = months; PA = physical activity; PDPAR = Previous Day Physical Activity Recall; RCT = randomised controlled trial
