# Supplementary figures and images for: The effectiveness and complexity of interventions targeting sedentary behaviour across the lifespan: a systematic review and meta-analysis
Source: Int J Behav Nutr Phys Act. 2020 Apr 25;17:53. doi: 10.1186/s12966-020-00957-0 (PMC7183680; doi:10.1186/s12966-020-00957-0)

**Risk of Bias: Adults (n=77) Risk of Bias: Children (n=84)**

**
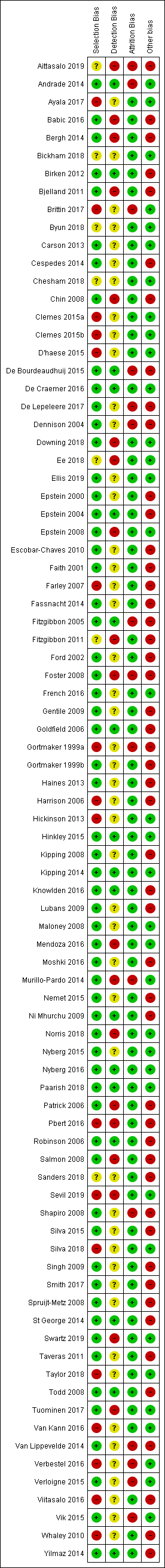

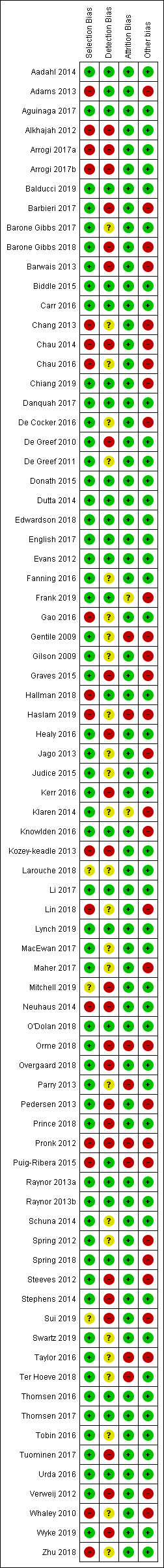
**

Supplement: Supplementary file 3 — Additional file 3: Supplement C. Risk of Bias [file 12966_2020_957_MOESM3_ESM.docx]
